# Navigation Simulation of a Mecanum Wheel Mobile Robot Based on an Improved A* Algorithm in Unity3D

**DOI:** 10.3390/s19132976

**Published:** 2019-07-05

**Authors:** Yunwang Li, Sumei Dai, Yong Shi, Lala Zhao, Minghua Ding

**Affiliations:** 1 School of Mechatronic Engineering, China University of Mining and Technology, Xuzhou 221116, China; 2 Department of Mechanical Engineering, Stevens Institute of Technology, Hoboken, NJ 07030, USA; 3 School of Mechanical and Electrical Engineering, Xuzhou University of Technology, Xuzhou 221018, China

**Keywords:** navigation simulation, path planning, improved A* algorithm, Unity3D, Mecanum wheel robot

## Abstract

Computer simulation is an effective means for the research of robot navigation algorithms. In order to implement real-time, three-dimensional, and visual navigation algorithm simulation, a method of algorithm simulation based on secondary development of Unity3D is proposed. With this method, a virtual robot prototype can be created quickly with the imported 3D robot model, virtual joints, and virtual sensors, and then the navigation simulation can be carried out using the virtual prototype with the algorithm script in the virtual environment. Firstly, the scripts of the virtual revolute joint, virtual LiDAR sensors, and terrain environment are written. Secondly, the A* algorithm is improved for navigation in unknown 3D space. Thirdly, taking the Mecanum wheel mobile robot as an example, the 3D robot model is imported into Unity3D, and the virtual joint, sensor, and navigation algorithm scripts are added to the model. Then, the navigation is simulated in static and dynamic environments using a virtual prototype. Finally, the navigation tests of the physical robot are carried out in the physical environment, and the test trajectory is compared with the simulation trajectory. The simulation and test results validate the algorithm simulation method based on the redevelopment of Unity3d, showing that it is feasible, efficient, and flexible.

## 1. Introduction

Navigation is one of the most challenging competencies required of an autonomous mobile robot (AMR). It can be defined as the combination of the four fundamental competences: perception, localization, path planning, map building, and interpretation. Robot navigation refers to the robot’s ability to determine its own position in the environment and then to plan a path towards its goal positions based on its knowledge about the environment and sensor values so as to reach its goal positions as efficiently and reliably as possible. Research on navigation algorithms is necessary to improve automatic mobile robots in all fields [1,2,3,4,5,6]. The navigation algorithm is the key technology for the autonomous navigation of robots, and it is also a research hotspot in the field of AMR. The use of modeling and simulations to develop navigation algorithms provides development flexibility and the capability to conduct extensive testing of the algorithm under a variety of operational environments and robot configurations [7]. So, algorithm simulations have been widely used in navigation algorithm research. 

Previously, the algorithm simulation of robots was usually developed using C++, Java, MATLAB and other programing languages. In the study presented in [8], an algorithm for path planning to a target for a mobile robot in an unknown environment was implemented in Borland C++; afterwards, it was tested with Visual Basic and DELPHI programming language. The motion of the robot that moves from the initial position to the desired position following an estimated trajectory was shown in the simulation. In the study presented in [9], a path finding simulator for the Pioneer 3DX mobile robot was designed with GUI (Graphical User Interface) in MATLAB. Five different algorithms, including the Dijkstra algorithm and A* algorithm, were implemented to determine the shortest path for a mobile robot between nodes within various mazes using the simulator. Using this simulation method, usually only two-dimensional simulation results can be displayed, and the display effect is not good. In order to show the results of the algorithm better, sometimes the navigation algorithm is simulated jointly by using programming language such as MATLAB and dynamic simulation software such as ADAMS, RecurDyn. In the study presented in [10], the performance of an adaptive impedance algorithm for tendon-driven dexterous hands was validated by using MATLAB and ADAMS software in a joint simulation. A tendon-driven hand model was built and a control module was generated in ADAMS. Then, the control system was built in MATLAB using the control module. However, the simulation speed of this method is not very fast, and the simulation parameters can only be set in advance, so the real-time performance of the simulation is poor. In the simulation of some robots, especially industrial robots [11,12,13], the position coordinates of each part of the robot are calculated by programming language, such as C++, and the corresponding 3D graphics are drawn by OpenGL to display the corresponding calculation results dynamically. This kind of three-dimensional motion simulation only shows the positions of the robot parts calculated by the kinematics equation, not by the physical engine. It is usually used in the simulations of industrial robots, but it is difficult to simulate some mobile robots with higher physical effect requirements using this method.

At present, there are also several commercial, professional robotic simulation platforms including software such as Webots, MRDS, Gazebo, MORSE, V-REP, Simbad, USARSim, STDR/Stage, and ARGoS [14,15,16,17,18,19]. Webots, an open-source robot simulator, can model and simulate any mobile robot, including wheeled, legged, and flying robots. It includes a complete library of sensors and actuators, and can be programmed in C, C++ and Java, or from third party software through TCP/IP [20]. By using Webots, virtual environments can be achieved for robot simulations. Additionally, Webots allows 3D models that use the VRML97 standard to be imported [21]. MRDS (Microsoft Robotics Developer Studio) [22], a visual programming tool, is a Windows-based environment for robot control and simulation. MRDS not only supports the visual programming language, but also supports many programming languages such as Visual Basic, Visual C++, and IronPython. MRDS’s Visual Simulation Environment (VSE) ensures a high quality simulated environment by using NVIDIATM PhysXTM to create high fidelity 3D simulations with realistic object interactions [23]. Gazebo [24], a well-designed simulator, makes it possible to rapidly test algorithms, design robots, perform regression testing, and train AI systems using realistic scenarios. Gazebo offers the ability to accurately and efficiently simulate populations of robots in complex indoor and outdoor environments. It supports multiple physics engines (ODE, Bullet, DART). The graphic engine is robust and ensures the development of high-quality 3D models. It has been widely used in robot and multi-robot system simulations [25,26,27] Gazebo is mainly used in the Linux system. Although we can set up a workspace for compiling Gazebo on Windows, it does not work in a very stable condition on Windows at present. V-rep (Virtual Robot Experimentation Platform) is a general purpose robotic simulator with an integrated development environment developed by Coppelia Robotics [28]. V-rep supports many programming languages and has three graphical engines to compute faster dynamics and to simulate physics and object interactions. It is commonly used for the navigation simulation of robots [29,30,31]. MORSE (Modular Open Robots Simulation Engine) [32] is based on the open-source project Blender, a 3D game engine that comes with an integrated bullet physics engine. MORSE operates from a command line, and it is a purely Python application that supports almost any 3D model. Simbad, a 3D robot simulator, is a simple testing platform to study artificial intelligence and AI algorithms for autonomous robots and agents. However, this simulation tool does not provide a realistic simulation of the robot environment [33]. USARSim (Unified System for Automation and Robot Simulation) [18] is a 3D simulator based on the Unreal Tournament (UT) game engine. USARSim was developed to simulate multiple robots in search and rescue environments. It supports sound sensors, touch sensors, lasers, odometry, and cameras. Sim2Real (simulation to reality), which tends to be photo-realistic, is a hotspot in the research and application of robot simulations at present. In the study presented in [34], the use of LiDAR sensor modeling and data augmentation with GANs for autonomous driving was studied. CycleGANs was employed to solve the sensor modeling problem for LiDAR to produce realistic LiDAR data from a simulated LiDAR (sim2real). In the study presented in [35], Sim2Real viewpoint invariant visual serving by recurrent control was studied. The paper describes how the resulting model can be transferred to a real-world robot by disentangling perception from control and only adapting the visual layers. The ROS (Robot Operating System) is an open-source, meta-operating system for robots, and it is one of most popular types of robotics middleware. It currently only runs on Unix-based platforms. Rviz (ROS visualization) is a 3D visualizer for displaying sensor data and state information from ROS. Using Rviz, the current configuration on a virtual model of the robot can be visualized, and the live representations of sensor values coming over ROS topics can also be displayed. ROS is widely used in robot control and algorithm simulations. In order to better simulate the algorithms and display the simulation results, ROS is usually combined with Gazebo and Rviz [15,36,37]. 

Unity3D is a game development platform. It is a fully integrated professional game engine and also has a variety of inbuilt user interfaces and 3D rendering capabilities alongside its own networking protocol [38]. Unity3D makes use of programming language and its own development environment to create attractive 3D games and software. Unity3D also has a good simulation function for kinematics and dynamics based on the physical engine, due to the integration of PhysX, which is a scalable multi-platform game physics solution. In addition to game development, Unity3D has been applied in a wide range of fields [39,40], such as virtual places, visualization building, virtual teaching and training, and machine motion simulation. Unity3D is also used in robot kinematics, dynamics, and navigation algorithm simulations. In paper [41], the method of robot simulation using the graphics engine and physical engine of Unity3D is shown. This method obtains realistic simulations of the execution of robotic tasks including sensing and motion primitives. Paper [42] presents the implementation of a Unity3D-MATLAB simulator applied to the area of robotics. In the simulator, Unity3D exchanges information with MATLAB to execute different proposed control algorithms. In paper [43], a ROS-Unity3D based system is introduced for the monitoring of an industrial robotic process as well as a framework to simulate and execute an industrial process monitoring task in Unity3D. In paper [44], a novel real-time three-dimensional simulation system, ROSUnitySim, is presented using ROS and Unity3D, for local planning by miniature unmanned aerial vehicles (UAVs) in cluttered environments. The paper particularly introduces the modeling of environments and LiDAR sensor. In the study presented in paper [45], the 3D shortest distance was studied using the A* algorithm in Unity3D. The applications of Unity have also been extended to machine learning. The Unity Machine Learning Agents Toolkit (ML–Agents) is an open-source Unity plugin for creating and interacting with simulation environments using the Unity platform. By taking advantage of Unity as a simulation platform, the toolkit enables the development of learning environments which are rich in sensory and physical complexity, provide compelling cognitive challenges and supporting dynamic multi-agent interactions [46]. In paper [47], in order to train and evaluate interactive agents in realistic simulated environments, the Interactive Question Answering Dataset (IQUAD V1), which builds on AI2-THOR [48], a photo-realistic customizable simulation environment for the integration of indoor scenes with the Unity physics engine, is presented. 

The types of professional robotic simulation software mentioned above each have their own advantages and disadvantages. Researchers need to make reasonable choices based on their actual needs. Some researchers have also analyzed and compared these simulators [15,17,19,49], as shown in Table 1, which can provide references for making choices. The above professional robot simulation platforms or simulators have their own characteristics and have been used widely. Users can choose between them according to their own unique needs. However, many of them provide either unrealistic visual information, inaccurate physics, low task complexity, or a limited capacity for interactions among artificial agents [47]. Since Unity3D has many advantages mentioned above, it is a good idea to implement the navigation algorithm simulating autonomous mobile robots, taking advantage of the realistic interactions between the robot and all the other elements of the environment that Unity3D provides. In this paper, through the secondary development of Unity3D, a navigation simulation platform based Unity3D is designed. Using the simulation platform, a virtual robot prototype can be established quickly with the imported 3D robot model and virtual joints and sensors, and navigation algorithm scripts can be added to the virtual prototype to carry out navigation simulations in the virtual ground environment. In this paper, the A* algorithm was improved for navigation in unknown 3D environment. Taking the Mecanum wheeled mobile robot as an example, its 3D robot model was imported into Unity3D, and the joint, sensor and navigation algorithm scripts are added to the model, and then the improved A* navigation algorithm was simulated using the robot virtual prototype. The test was carried out using the physical robot prototype in the physical environment, and the simulation trajectory and test trajectory were compared to verify the feasibility and availability of the proposed method.

This paper is organized as follows: In Section 2, the simulation platform based on Unity3D is described, and the creation of the virtual joints, sensors, and environments in Unity3D is introduced. An improved A* algorithm that can be used in an unknown 3D environment is introduced in Section 3. Section 4 firstly describes the Mecanum wheel mobile robot and its kinematics model and then introduces the virtual prototype of the robot and the navigation simulation process of the improved A* algorithm on the simulation platform. In Section 5, a navigation accuracy measurement experiment system for robots in the physical environment is created to evaluate the simulation effect of the simulation platform created in Unity3D, and the test results of physical robot in the physical environment are compared with the simulation results.

## 2. Simulation Platform Based on Unity3D

### 2.1. Elements of the Simulation Platform

The elements of the simulation platform include the mobile robot virtual prototype, a simulation environment for the prototype, a kinematic model, virtual sensors, a virtual actuator, a graphics engine, a physics engine, and a navigation algorithm. The mobile robot virtual prototype, which is an autonomous entity with actuation and sensing capabilities, is the main element of the simulation platform [41]. The 3D model of the mobile robot can be modeled using any 3D modeling tool, such as SolidWorks, and then it can be imported into Unity3D. The mass characteristics should be added to the 3D model, and the actuation should be imposed on its mobile mechanisms, such as its wheels. The virtual sensors should be imported to the virtual prototype to retain knowledge about the prototype itself and about the environment. The virtual terrain environment includes ground features and obstacle features. In the simulation process, the mobile mechanism of the virtual prototype interacts with the ground features, such as uneven ground, slope, ditch, etc. The virtual sensor detects the entity features of virtual obstacles, such as rocks, and carries out path planning according to the navigation algorithm.

The virtual robot prototypes and terrain environment in the robot simulation platform based on Unity3D will change according to the needs of different robot simulations. Different virtual prototypes of robots have different mechanical structures and sensor configurations. In order to build virtual prototypes efficiently, it is necessary to create parametric kinematic joints and parametric sensor modules. The location of various types of obstacles and the ground features in the simulation environment will also change. Therefore, in order to improve the efficiency of the creation of a virtual terrain environment, parametric programming should be adopted.

### 2.2. Parametric Virtual Kinematic Joints

There are many kinds of mechanisms in the mechanical body of a mobile robot. Kinematic joints (or simply, joints) are critical parts of a mechanism, which is a connection between two components of the mechanism that imposes constraints on their relative movement. The types of motion allowed and constrained are related to the characteristics of the mechanism, which are usually characterized by the degrees of freedom it allows. In Unity3D, the configurable joint component can limit the degrees of freedom of relative motion between two components. Configurable joints are extremely customizable since they incorporate all the functionality of the other joint types. They can be used to create anything from adapted versions of the existing joints to highly specialized joints. There are two primary functions that the configurable joint can perform: movement/rotation restriction and movement/rotation acceleration. These functions depend on a number of interdependent properties. Restriction can be specified per axis and per motion type. The translation along an axis can be defined as “X Motion”, “Y Motion”, and “Z Motion”. The rotation around an axis can be defined as “Angular X Motion”, “Angular Y Motion”, and “Angular Z Motion”. Each one of these properties can be set to “Free” (unrestricted), “Limited”, or “Locked” (restricted to zero movement). By adding configurable joint components to the components of the joint and setting parameters, the degrees of freedom of the joints can be set, and the simulation of various simple joints, such as the revolute joint and the prismatic joint, can be realized. The virtual joints obtained by programming can be used to create virtual prototypes.

**Figure 1:**
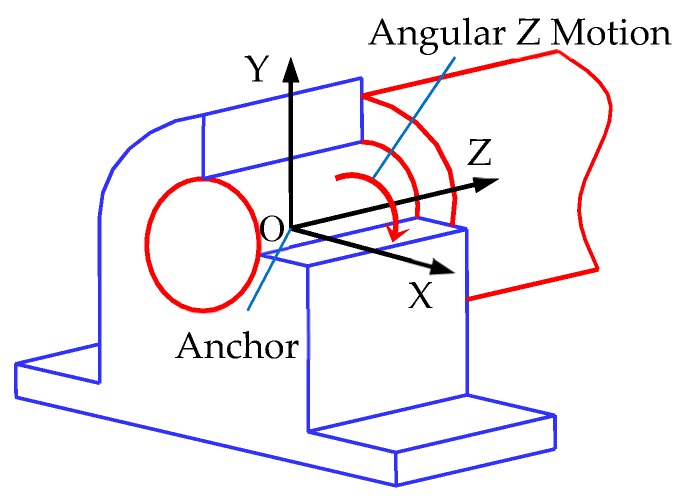
Schematic diagram of the revolute joint.

Taking the revolute joint as an example, the programming requirements of a parameterized virtual motion pair are introduced. The revolute joint is a kind of lower pair joint, which has one degree of freedom. In a three-dimensional coordinate system, the revolute joint can only rotate around a coordinate axis: X, Y, or Z. The degree of freedom of the revolute joint in Figure 1 is the degree of freedom around the Z axis. If a revolute joint rotating around the Z axis is created, as shown in Figure 1, “Angular Z Motion” should be set as “Free”, and “Angular X Motion” and “Angular Y Motion” should be set as “Locked”. The “Anchor”, which is the point where the center of the joint is defined, needs to be set. The “Position Damper” of the “Angular Z Drive” and “Connected Body” should also be set. The script of the revolute joint can be compiled according to the requirements of the revolute joint.

### 2.3. Virtual Sensors

Autonomous mobile robots need to be equipped with enough internal sensors and external sensors to respectively detect the internal state and external environmental information of the robot. Common internal sensors are motion output sensors such as encoders and inertial navigation systems. Common external sensors include ultrasonic sensors, laser ranging sensors, and 2D and 3D LiDARs. Only when these sensors are simulated can the robot detect its own motion state and the simulation environment in Unity3D, so as to realize the autonomous control of the robot.

Physics.Raycast in Unity3D can help to simulate the ranging sensors. The function of Physics.Raycast is to cast a ray of maxDistance length from an origin point in a specified direction against all colliders in the scene. This ray returns detailed information on what is hit. 

#### 2.3.1. Two-Dimensional LiDAR

**Figure 2:**
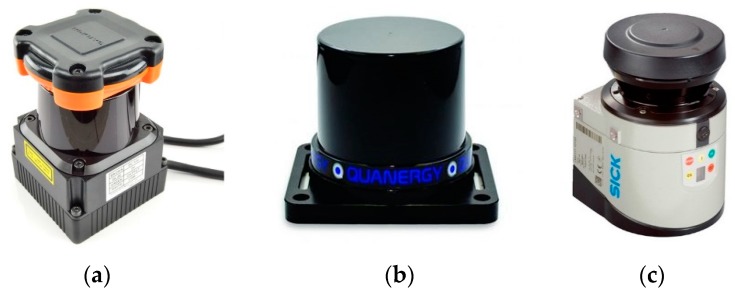
Common 2D LiDAR photos: (**a**) Hokuyo Utm-30LX scanning laser rangefinder LiDAR; (**b**) Quanergy's M8 LiDAR sensor; (**c**) SICK’s LMS151 field detection laser scanner

The LiDAR/LADAR/Laser radar, an instrument for laser detection and ranging, can cast a short, pulsed laser to a target object and then time how long it takes for the light to return. LiDAR can also provide an image of the target at the same time as determining the distance. The common types of 2D LiDAR are shown in Figure 2. Based on the characteristics of 2D LiDAR, we used the Physics.Raycast function in Unity3D to simulate it.

**Figure 3:**
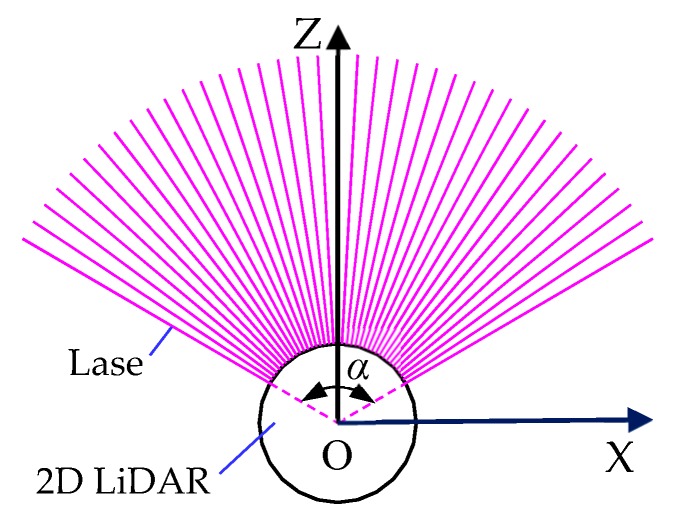
Schematic diagram of 2D LiDAR detection.

**Figure 4:**
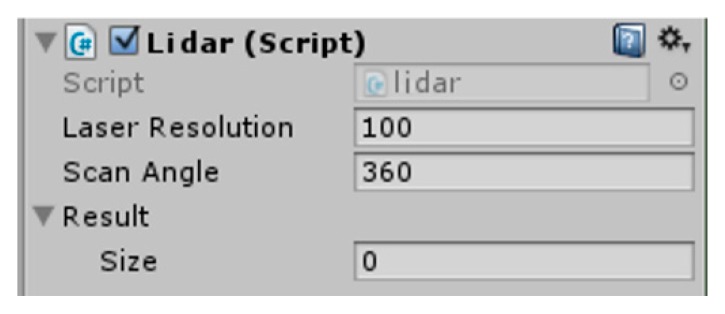
Programming page of the 2D LiDAR script.

Firstly, variables for scripting are defined in Table 2 according to parameters of Physics.Raycast. As shown in Figure 3, angle α is the value of scanAngle, and the angular bisector line of angle α is the Z-axis direction of the LiDAR. The total number of laser lines in the range of angle α is the value of laserResolution. Detection starts from the left-most laser line and proceeds to the right. First, the radar rotates around the angle of scanAngle/2 to the left, and then it uses the Physics.Raycast function to transmit a ray to the front. The result is stored in the result array. Next, the LiDAR rotates around the deltaAngle angle to the right and casts another laser. The result is stored in the result array until all the laser lines have been detected. Finally, the laser rotates the angle of the scanAngle to the left to prepare for the next scan detection. Thus, the data stored in the result array are the detection results of the LiDAR. Each value in the array is the distance from the obstacle detected by the left-to-right laser line to the radar. If the value is–1, there is no the obstacle on the laser line. The distance to each obstacle around the radar is obtained by reading the result array. Using this method, the script of 2D LiDAR simulation can be compiled. In Unity3D, the page displayed after addition of the above 2D LiDAR script is shown in Figure 4. After clicking the Play button of Unity3D, the distance values measured by the function are displayed on the result array.

#### 2.3.2. Three-Dimensional LiDAR 

**Figure 5:**
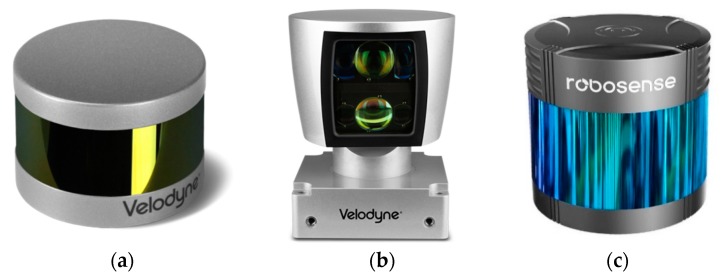
Three-dimensional LiDAR photos: (**a**) Velodyne VLP-16 LiDAR; (**b**) Velodyne HDL-64E LiDAR; (**c**) RoboSensep’s RS-LiDAR-32.

The common type of 3D LiDAR is shown in Figure 5. The main difference between 3D LiDAR and 2D LiDAR is that 3D LiDAR casts multiple laser lines in order to realize multi-layer detection. In Unity, 3D LiDAR is also simulated using Physics.Raycast. A two-dimensional array can be used to store the detection return value. The definitions of variables for 3D LiDAR simulation are shown in Table 3.

The simulated 3D LiDAR here refers to IBEO’s 4-line and 8-line 3D LIDAR and Velodyne’s 16-line, 32-line and 64-line 3D LiDAR. The values of deltaLineAngle and maxLineAngle are shown in Table 4, which are determined by the type and number of lines of the LiDAR.

**Figure 6:**
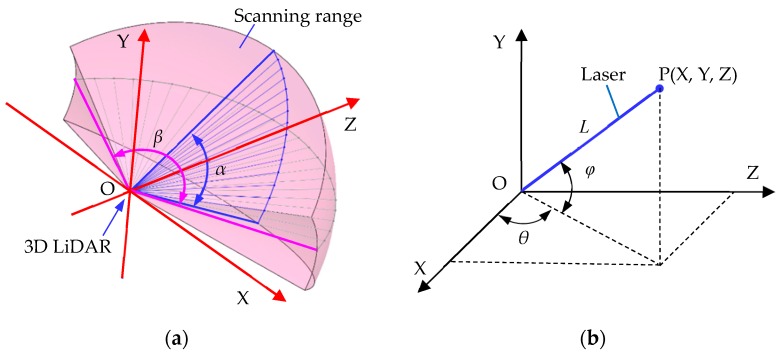
Schematic diagram of 3D LiDAR detection: (**a**) the scanning range of the 3D LiDAR; (**b**) schematic diagram of the radar detection results transformation.

As shown in Figure 6, angle α in Figure 6a represents the scanning range of the 3D LiDAR in the vertical direction. The total number of laser lines in the vertical direction is the lineNumber. The angular bisector of angle α is the Z-axis direction of the radar. Angle *β* in Figure 6a is the horizontal scanning range of 3D LiDAR, that is, the value of scanAngle. The total number of laser lines in the range of angle *β* is the value of laserResolution. As shown in Figure 6b, taking a laser line as an example, *L* is the distance from the LiDAR to the collision point; angle *φ* is the angle between the laser line and the horizontal plane, angle α can be obtained by the maxLineAngle and deltaLineAngle; and angle *θ* is the angle between the laser line and the vertical plane in front of the radar and is obtained by the scanAngle and deltaAngle. The 3D vectors of the collision point relative to the LiDAR can be obtained by the following formula:

$$\begin{array}{c} \left[\begin{array}{c} x\\ y\\ z \end{array}\right] \end{array}=\left[\begin{array}{c} \cos\varphi \cdot \cos\theta \\ \sin\varphi \\ \cos\varphi \cdot \sin\theta \end{array}\right]\cdot L$$

**Figure 7:**
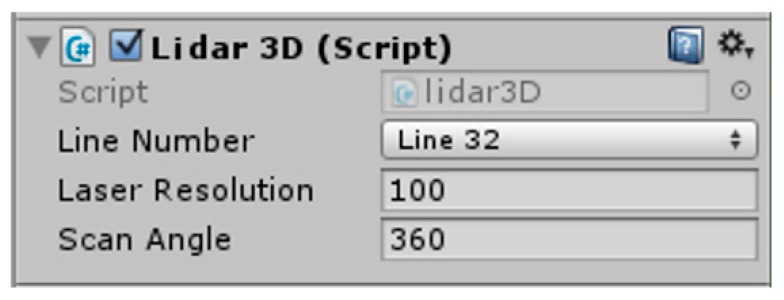
Program page of the 3D LiDAR script.

The program page for adding a 3D LiDAR script to the components in Unity3D is shown in Figure 7. 

### 2.4. Construction of Virtual Simulation Environment

When the virtual prototype of a robot is simulated in Unity, it is necessary to create virtual environments to simulate the real environments. When building a simulation environment, it is necessary to simulate the ground and various other obstacles so that the motion state of the robot can be observed when it passes through obstacles. This section briefly introduces the creation of stochastic ground and parameterized obstacles.

#### 2.4.1. Stochastic Ground Simulation

In Unity, Terrain is the most important component for building the terrain environment. Using the HeightMap parameter option in Terrain, ground can be constructed by importing an image in RAW format. The gray scale of each pixel in the RAW image corresponds to the height of each position in Terrain. Thus, when constructing stochastic ground, we can first generate a gray image corresponding to the height of each location of the ground by writing a program, then convert the image into RAW format, and finally, import the RAW image into the Terrain component to generate a random ground surface.

Usually, the power spectrum of pavement irregularity is used to express the magnitude of random pavement irregularity. For the 3D modeling of stochastic ground or road, many methods can be used such as the white noise method, the Fast Fourier Transform (FFT) method, and the harmonic superposition method. This section describes the selection of the sine wave superposition principle and the writing of the program in C# language to calculate the unevenness distribution of the pavement, thus converting the pavement height to generate the corresponding three-dimensional random pavement. The basic principle of the sinusoidal wave superposition method is as follows:

For the spatial frequency $n_{1}<n<n_{2}$, the variance of ground roughness $\sigma_{d}^{2}$ can be obtained from the power spectral density of ground roughness $G_{d}\left(n\right)$, and the formula can be expressed as

$$\sigma_{d}^{2}=\int_{n_{1}}^{n_{2}}G_{d}\left(n\right)d$$

The power spectral density of ground roughness $G_{d}\left(n\right)$ can be obtained from the Chinese national standard GB7031-86, and the fitting expression is as follows:

$$G_{d}\left(n\right)=G_{d}\left(n_{0}\right){\left(\frac{n}{n_{0}}\right)}^{-W}n>0$$

where,$\;n_{0}$ references the spatial frequency, generally taken as $n_{0}=0.1\;m^{-1}$.

W is Frequency index that determines the frequency structure of the pavement power spectral density, generally taking W=2;

In the integral operation, the spatial frequency $n_{1}<n<n_{2}$ can be divided into m intervals with widths of $\Delta n_{i}$. This is replaced by the power spectral density of pavement roughness $G_{d}\left(n_{mid,i}\right)$ at the center frequency $n_{mid,i}$ (i=1,2,⋯,m) of each cell, and the variance of pavement roughness $\sigma_{d}^{2}$ is obtained by the discrete method, and the formula can be changed to $G_{d}\left(n_{mid,i}\right)\Delta n_{i}$

$$\sigma_{d}^{2}=\overset{m}{\underset{i=1}{\sum }}G_{d}\left(n_{mid,i}\right)\Delta n_{i}$$

So, we can use the sinusoidal wave function to represent the ground surface model and get the Stochastic ground surface roughness. The spatial frequency of the sinusoidal wave function is $n_{mid,i}$ (i=1,2,⋯,m), the standard deviation is, $\sqrt{G_{d}\left(n_{mid,i}\right)\Delta n_{i}}$ ,and the formula of the sinusoidal wave function is

$$q_{i}\left(x\right)=\sqrt{2G_{d}\left(n_{mid,i}\right)\Delta n_{i}}\sin\left(2\pi n_{mid,i}x+\theta_{i}\right)$$

By superposing the sinusoidal wave functions of m intervals, the model of random pavement roughness can be obtained. The formula is as follows:

$$q\left(x\right)=\overset{m}{\underset{i=1}{\sum }}\sqrt{2G_{d}\left(n_{mid,i}\right)\Delta n_{i}}\sin\left(2\pi n_{mid,i}x+\theta_{i}\right)$$

$\theta_{i}$ is a random number belonging to [0, 2π].

The above formula represents a longitudinal unevenness distribution of the ground surface. For 3D space, it is necessary to obtain the longitudinal and lateral ground surface irregularities of the ground surface. The lateral roughness model of the pavement is the same as that for the longitudinal direction. After the same discrete process, the ground roughness in 3D space can be obtained:

$$q\left(x,y\right)=\overset{m}{\underset{i=1}{\sum }}\sqrt{2G_{d}\left(n_{mid,i}\right)\Delta n_{i}}\sin\left(2\pi n_{mid,i}x+\theta_{i}\left(x,y\right)\right)$$

where $\theta_{i}\left(x,y\right)$ is a random number belonging to [0, 2π].

After obtaining the ground roughness formula in 3D space, the program can be written in C# language to generate the corresponding grayscale image and then saved in PNG format. After Gaussian blur processing, the image is saved in RAW format. Finally, the RAW format image is imported and converted to 3D terrain using the Terrain component in Unity software.

**Figure 8:**
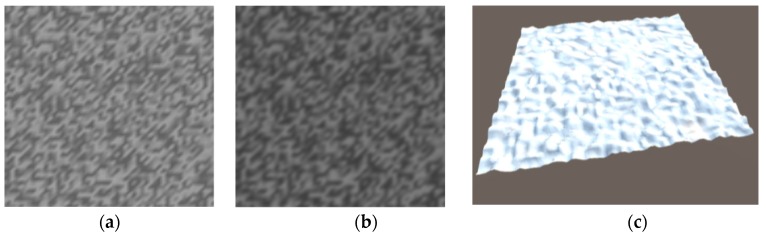
Using the script to create stochastic ground: (**a**) PNG image; (**b**) PNG image after Gauss blur; (**c**) stochastic ground after Gauss blur.

Figure 8a shows the PNG image generated by the program, Figure 8b shows the PNG image of the Gus fuzzification process, and Figure 8c shows the terrain created in Unity3D from the RAW image converted from Figure 8b.

#### 2.4.2. Simulation of Parameterized Obstacles

**Figure 9:**
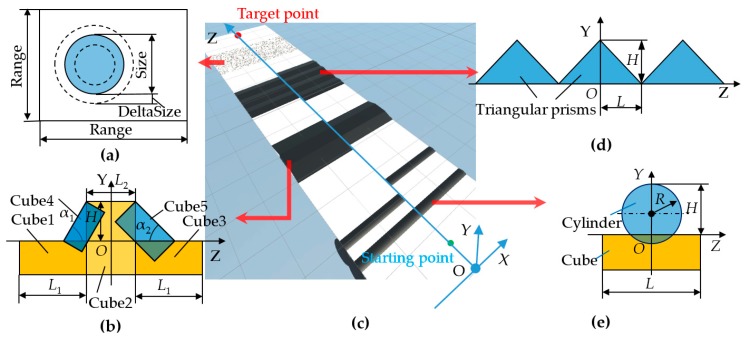
Test terrain platform built using multiple modules: (**a**) scattered gravel pavement; (**b**) slope step; (**c**) test terrain platform; (**d**) undulating ground; (**e**) round convex obstacle.

In the simulation environment, it is necessary to extract and simplify the features of real obstacles such as the slope, step, channel, convex terrain, and so on. In the obstacle simulation, the slope step can be considered to be composed of five cubes, the undulating ground is composed of several triangular prisms, and the round convex terrain is composed of a cylinder and a cube, as shown in Figure 9b,d,e, respectively. The scattered gravel pavement can also be automatically created by parameterization, as shown in Figure 9a. According to the values of the parameters in Table 5, a test terrain platform with a length of 15,000 mm and a width of 4000 mm is built, as shown in Figure 9c. In this paper, the script of obstacle simulation is not introduced in detail.

#### 2.4.3. Virtual Environments for Navigation Simulation of the Robot

**Figure 10:**
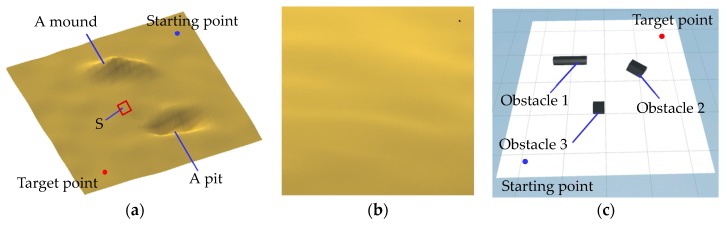
Simulation environments for the robot: (**a**) stochastic ground with a pit and a mound; (**b**) an enlarged view of the area S of the stochastic ground; (**c**) a planar ground with three obstacles.

Several different simulation grounds and obstacles were created to simulate the experimental navigation environment for a robot. A stochastic ground was created by using the stochastic ground surface generation program, and a pit and a mound obstacle terrain which could not be passed by robots were added by the adjustment function of the Terrain component in Unity, as shown in Figure 10a. There were many small folds on the stochastic ground. Figure 10b is an enlarged view of an area of the stochastic ground in Figure 10a. A planar ground with three obstacles was also created, as shown in Figure 10c. 

## 3. Improved A* Algorithm 

Path planning is the task of finding a continuous path that will drive the robot from the start point to the target point. Based on the information about the obstacles, the working environment of a robot can be categorized as a completely known environment, a partially known environment, or a completely unknown environment. It can also be categorized as a static environment or a dynamic environment [50,51,52]. There are many path planning and navigation algorithms, such as PRM, RRT, EST, RRT*, APF, MPC, ANN, GA, PSO, ACO, and D* [53], compared to which the A* algorithm has advantages such as its simple principles, easy realization, and high efficiency. Thus, it has been widely investigated and applied. To increase the applicability of the A* algorithm, meet the requirements for navigation tasks, generate more smooth paths, and reduce the length and turning times, many improved A* algorithms have been proposed and studied in depth. A 3D A* algorithm was studied to configure the path between two nodes in a 3D environment, and was shown to be faster than an A* Algorithm with 2D layers [45]. An improved A* algorithm was studied to improve the safety and smoothness of the planned path and to reduce the movement time of the robot in complex terrain [53]. Several modifications (Basic Theta*, Phi*) and improvements (RSR, JPS) of the A* algorithm have been studied to reduce the computational time and optimize the path optimality [54]. A modified A* algorithm for path planning with efficient coverage was presented, and can be used to generate waypoints in order to cover the narrow spaces [55]. An improved A* algorithm considering water current, traffic separation, and berthing for vessel path planning [56], which achieves the trade-off between path length and navigation safety, was proposed. So, the A* algorithm has good expansibility and adaptability and can be improved according to the actual working environment of the robot. Although the research interest of this paper is to propose a new method of navigation algorithm simulation in Unity3D, the research focus is not on the algorithm itself. In order to verify the availability and reliability of the simulation platform based on Unity3D, it is necessary to select the appropriate robot prototype and navigation algorithm. Therefore, the A* algorithm was selected to test and study the robot navigation simulation proposal.

### 3.1. Introduction of the A* Algorithm 

The A* search algorithm is a global optimization and state space heuristic algorithm. It can be seen as an improved version of the Dijkstra algorithm with the addition of an evaluation function [57]. In the search process, each search position in the state space is evaluated, and the least evaluated position is selected. Then, the search is carried out from this location until the target point is found. This can omit a large number of invalid search paths and improve the efficiency.

**Figure 11:**
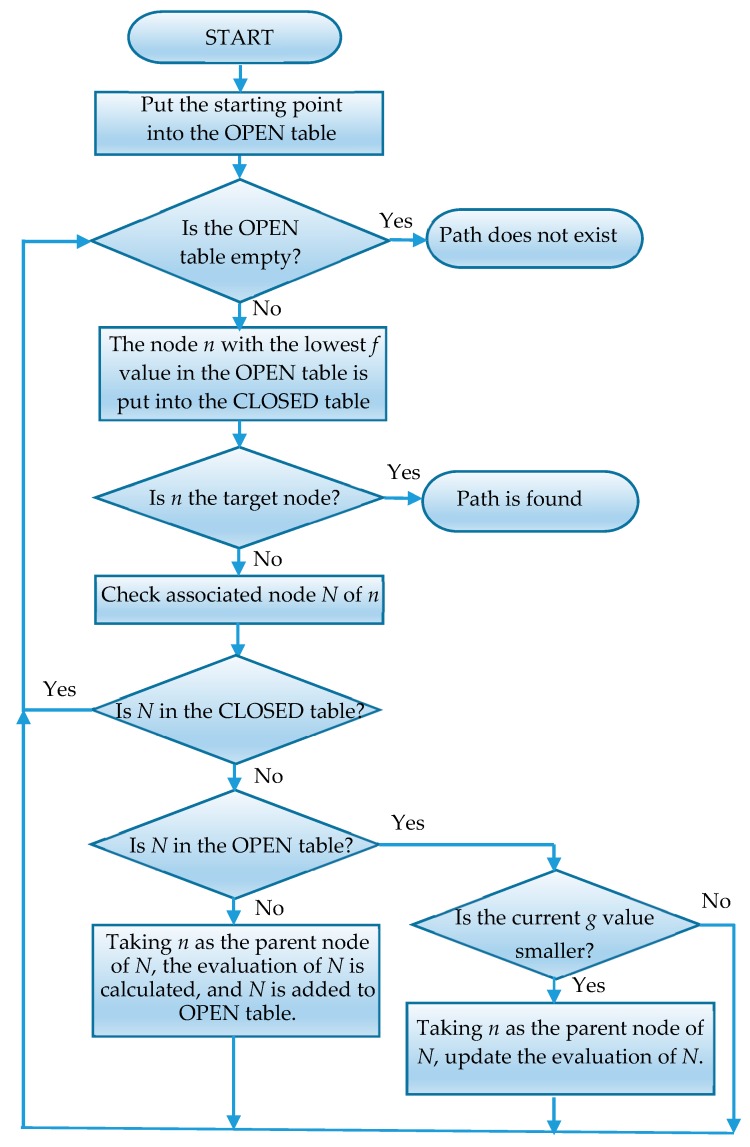
A* algorithm flowchart.

The evaluation function of the A* algorithm is as follows:

f(n)=g(n)+h(n)

where *f*(*n*) is the estimated cost of arriving at the target node from the initial node through node *n*. *g* (*n*) is the actual cost for travelling from the initial node to node *n* in the state space. *h* (*n*) represents the cost of estimating the optimal path from node *n* to target node. When the evaluation cost *h* (*n*) is closer to the real value, the efficiency of the algorithm is higher, and the likelihood of finding the optimal solution is higher. The flow chart of the A* algorithm is shown in Figure 11.

### 3.2. Improvement of the A* Algorithm for Navigation in an Unknown Environment

The A* navigation algorithm is a global path planning algorithm for use in known environments. When a mobile robot navigates in an unknown environment, it needs to move while detecting and planning the path in real time according to the terrain and obstacles detected. 

The strategy of the improved A* algorithm is to use the A* algorithm to conduct path planning in unknown environments and to make the mobile robot plan its movements. The robot continuously detects the surrounding environment in the course of movement and projects the detected environmental information into the map. If the detected obstacles do not block the planned path, the robot will continue to move along the original path. If the obstacles detected obstruct the planned path, the current position of the robot is set as the starting point of navigation, and the shortest path to the target point is re-planned according to the new environmental information at this time. On the whole, this path is not the shortest path from the original starting point to the target point, but it can avoid the roundabout path of the mobile robot.

### 3.3. Improvement of the A* Algorithm for Navigation in 3D Space 

**Figure 12:**
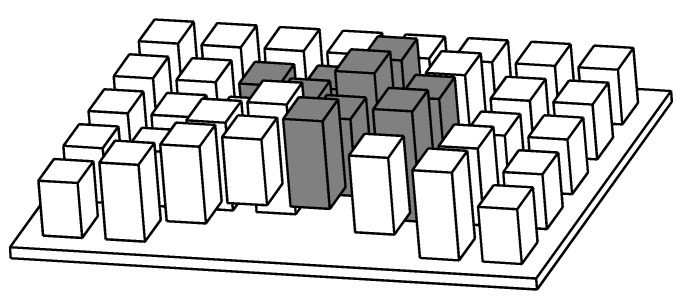
Schematic diagram of the improved node information.

The A* algorithm is suitable for path planning in 2D space and cannot be used directly for navigation in a 3D environment. For the A* algorithm, there are two types of storage information for each grid point, which are the location information of the node in 2D space and whether the node can be passed. In order to use the A* navigation algorithm in 3D space, two variables, maxHeight and minHeight, which store information in 3D space, need to be expanded to represent the maximum and minimum heights of nodes, respectively. As shown in Figure 12, the cuboids represent the nodes in the grid. Each node has its corresponding position information on the plane. The white cuboids represent the nodes through which the robot can pass, while the gray cuboids represent the nodes that the robot cannot pass through. The improved A* algorithm can record the height information of the nodes and realize the 3D map reconstruction, so that the robot can navigate in the 3D space.

**Figure 13:**
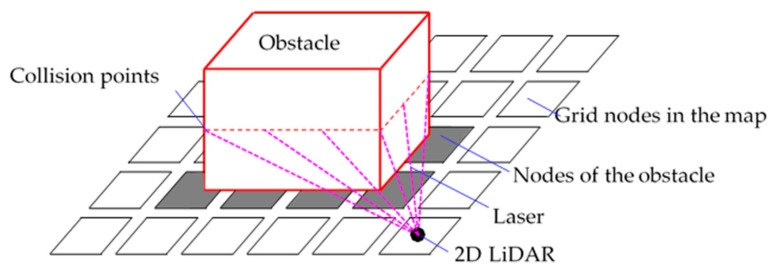
Obstacle judgment of the A* algorithm.

The A* algorithm is used to navigate in 2D space, and for the reconstruction of a 2D map, 2D LiDAR is used. As shown in Figure 13, 2D LiDAR casts laser lines, and when an obstacle is detected, the position information of the obstacle is projected into a 2D map, that is, the gray grid nodes, and the corresponding nodes of the obstacle are changed to be inaccessible.

**Figure 14:**
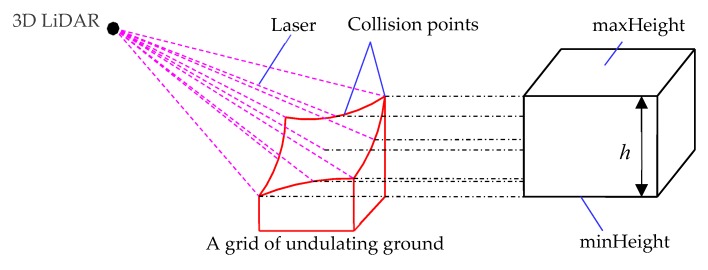
Schematic diagram of 3D LiDAR terrain detection.

**Figure 15:**
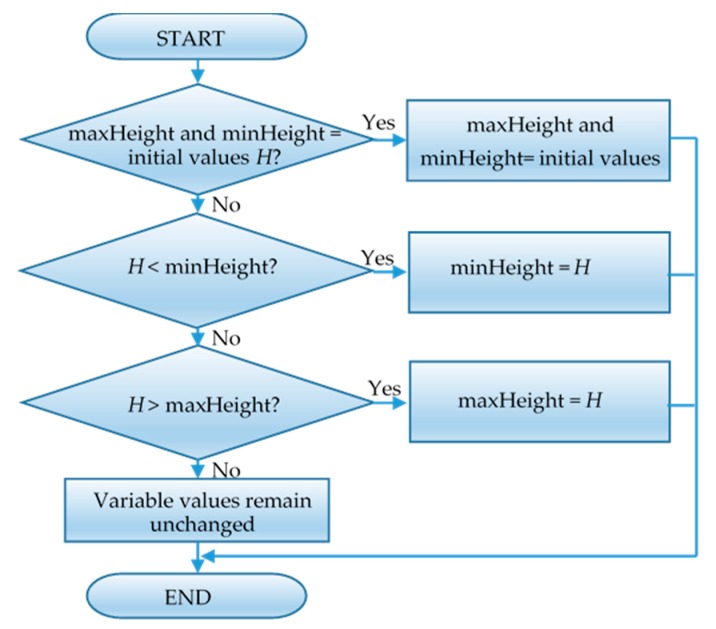
Node height information update flowchart (*H* = the height of the collision point).

When terrain reconstruction is performed in 3D space, the undulating ground and obstacles can be detected by 3D LiDAR. As shown in Figure 14, the irregular square is a block of undulating ground divided in a grid, The laser cast from a 3D LiDAR can detect all positions on the undulating ground, and all height information is updated to the variables of the node, As shown in the square on the right, the height of the top surface (maxHeight) of the square is the value of the node, and the height of the bottom surface (minHeight) is the value of the node. The height of the square is *h* (*h* = maxHeight-minHeight). Figure 15 shows how to update the node height information.

After 3D terrain detection, it is necessary to determine whether the robot can pass through a certain area according to the height information of the nodes. The first criterion is the height difference (maxHeight − minHeight) of the nodes, and the criterion is that the size of the grid is multiplied by a coefficient. The magnitude of this coefficient is determined by the obstacle-overcoming ability of the robot, which is highly related to the maximum obstacle that the robot can cross.

**Figure 16:**
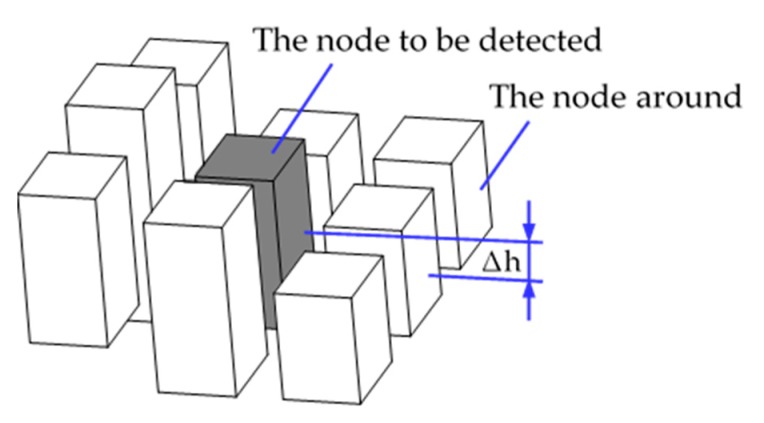
Schematic diagram to determine whether a node can pass through a certain area

The second criterion is the height difference between the node and the surrounding nodes. As shown in Figure 16, the intermediate gray blocks represent the nodes to be detected, and the eight white blocks represent the nodes around the nodes to be detected. The median height ((maxHeight + minHeight)/2) of the node to be detected is subtracted from the median height of the surrounding nodes, and the absolute value of the difference is used to determine whether the robot can pass through the node. The criterion is that the horizontal distance of the two nodes is multiplied by a coefficient. The coefficient is determined by the slope-climbing ability of the robot and is related to the maximum slope that the robot can climb.

### 3.4. Programming Implementation of the Improved A* Algorithm

The A* algorithm consists of four programs. The functions of the programs are to create nodes, to create grids, to calculate paths, to detect the environment, and to reconstruct maps. These are introduced separately.

#### 3.4.1. Creation of Nodes

The function of this program is to build a classification for nodes, which can directly generate objects of this class when creating each node in the grid. This simplifies the program and means that it does not need to be attached to Unity. It can be directly called by other programs. First, we define the name of the class as Node and then define the data members of the class, as shown in Table 6.

The way to get the target path from the parent variable is as follows: The target node is regarded as the first node of the path. The parent node of the target node is regarded as the second node of the path, and the parent node of the second node is regarded as the third node of the path until the starting node is found. The combination of the obtained nodes is the calculated target path. After the data member definition is finished, the constructor of the class is defined, and the four data members of the class, canWalk, worldPos, gridX, and gridY, are assigned values in the constructor.

#### 3.4.2. Creation of Grids

The function of this program is to divide the map into grids and create nodes at the intersections of grids. This program needs to be attached to Unity, and the objects in Unity are used to assign values to variables in the program. The data members for the Grid program are defined, as shown in Table 7.

The area size of the grids is determined by the starting position and the target position of the robot. The calculation method of the gridSize value used in this program is as follows:

gridSize.x = Mathf.Abs(endPoint.transform.position.x-robot.transform.position.x) * 1.5f;

gridSize.y = Mathf.Abs(endPoint.transform.position.z-robot.transform.position.z) * 1.5f.

After the data member definition has been completed, the member functions of the program are defined. The member function “CreatGrid” of the program is a program for dividing the grid, which runs when simulation begins in Unity. Firstly, the starting point of grid map is defined, which is the origin of the 2D map, where the starting point is selected in the lower left corner of the grid dividing area. The center point of the grid division area is calculated, and the coordinates of the center point are the average values of the coordinates of the starting position and the target position of the robot, that is, transform.position = (endPoint.transform.position + robot.transform.position)/2.

**Figure 17:**
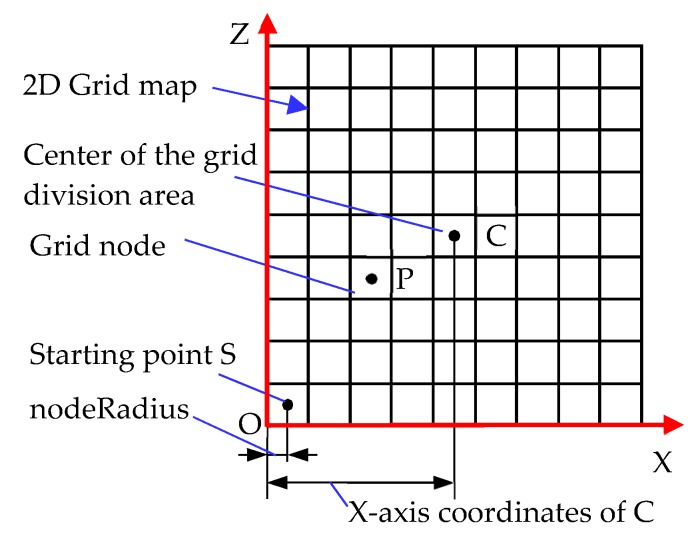
Schematic diagram of the dividing grid.

In Figure 17, Point C is the center of the grid division area, and Point S is the starting point of the grid map. The program for calculating coordinates of S point is as follows:

startPoint = transform.position − (gridSize.x/2 − nodeRadius) × Vector3.right − (gridSize./2 × nodeRadius) × Vector3.forward.

The other grid nodes in the map can be obtained from the starting point of the grid map plus a multiple of the node diameter, that is, the coordinates of the node in the 2D grid map. In the 2D grid map presented in Figure 16, the abscissa of point P is the abscissa of the starting point plus twice the diameter of the node, and the ordinate of point P is the ordinate of the starting point plus three times the diameter of the node. The corresponding calculation formula is

worldPoint = startPoint + Vector3.right × (2 × nodeDiameter) + Vector3.forward × (3 × nodeDiameter).

#### 3.4.3. Planning Path

The function of this program is to use the A* algorithm to calculate the shortest path from the starting position of the robot to the target position according to the map. This program needs to be attached to Unity, as some objects in Unity are used to assign values to some variables in the program. In addition, the above Grid program needs to be called.

A program named “Findpath” is defined, and then the data members of the program are defined, including robot, endPoint and grid, as shown in Table 8. After the data member definition has been completed, the member functions of the program are defined. A function is defined to assign the grid variables, that is, to call the Grid program. The assignment of variables uses the function grid = GetComponent<Grid>( ) in Unity. Next, a function is defined to calculate the path, and the calculated path is assigned to the path variable in the Grid program. The function first defines two variables of the List <Node> type: openSet and closeSet. Then, the program is written according to the flow chart of the A* algorithm shown in Figure 11.

If the number of elements in the openSet becomes zero, the path to the target point cannot be found; if the number of elements in the openSet is not zero, the following operation is performed: The node with the smallest fCost value in the openSet is moved into the closeSet. If this node is the target node at this time, then the path is found; otherwise, the calculation will continue. The nearest node of the current smallest node should be searched. If the nearest node is neither in the closeSet nor an obstacle, the current smallest node is set as the parent of the nearest node, the estimated value of the nearest node is updated, and the nearest node is added to the openSet.

#### 3.4.4. Environment Exploration and Topographic Reconstruction

**Figure 18:**
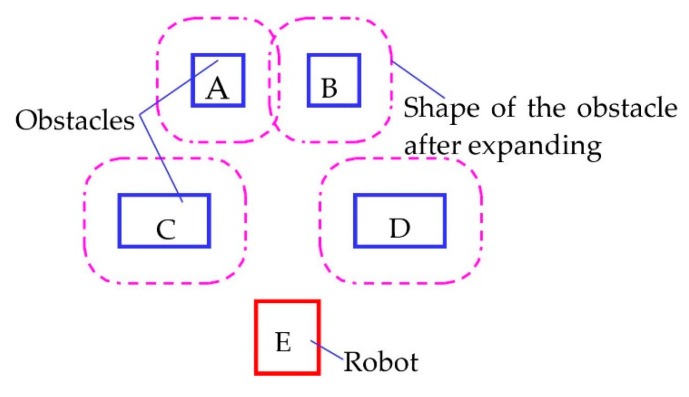
Schematic diagram of obstacle expansion.

The function of this program is to detect the environment by using 3D LiDAR and to create a 3D grid map. The path calculated by the “findpath” program drives the robot along the path to reach the target position. First, a program named “Navigation” is defined, and its data members and related variables are defined, as shown in Table 9. In the calculation of the path based on the A* algorithm, the robot is regarded as a particle, and the obstacle expansion method is used to obtain the path. The A, B, C, and D rectangles in Figure 18 represent the positions and sizes of the four obstacles, and rectangle E represents the size of the robot. The dotted line frame in the figure shows the shape of the obstacle after expanding the “barriderDistance” distance.

**Figure 19:**
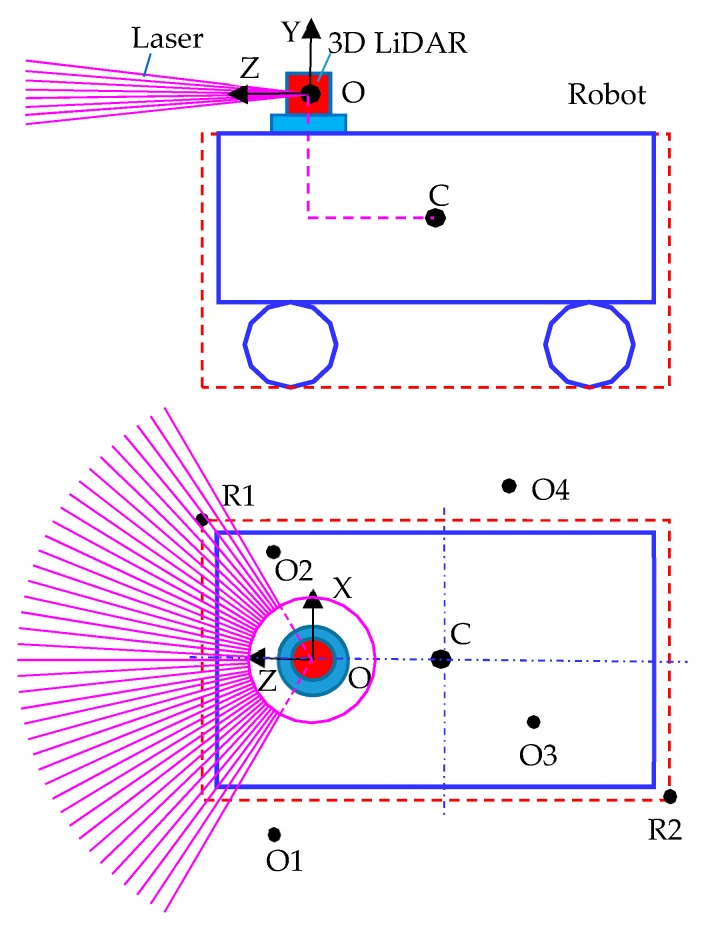
Schematic diagram of LiDAR position correction and detection results filtration.

As shown in Figure 19, Point C is the center position of the robot, that is, the current coordinate of the robot; Point B is the location of the LiDAR. The variable lidarPos stores three-dimensional vectors from point O to point C to correct the position of the robot from the radar position. The three-dimensional vectors of the collision point relative to point O measured by LiDAR and lidarPos can be used to obtain the three-dimensional vectors of the collision point relative to point C. Finally, two vector 3 variables RobSize1 and RobSize2 are defined. The variables store the size of the robot. When the LiDAR detects obstacles in this range, it is regarded as detecting the robot itself, ignoring the point automatically and preventing errors

**Figure 20:**
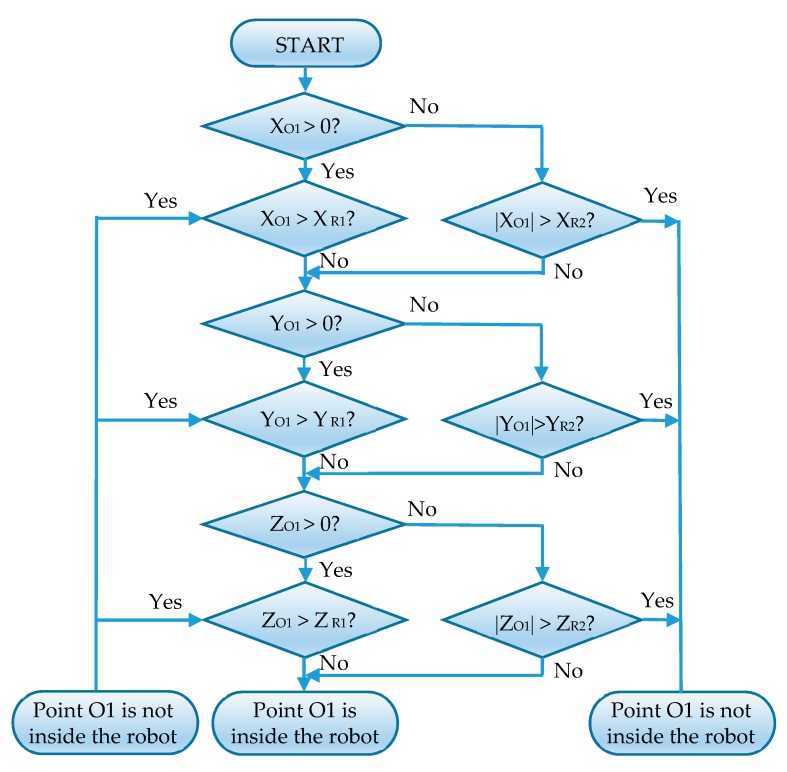
Detection results filtration flowchart.

In Figure 19, taking the overhead sketch of the robot as an example and taking the center O of the LiDAR as the origin, the shape and size of the robot are shown in the outermost solid box. Point R1 and point R2 correspond to the 3D vectors of the robotSize1 and robotSize2 variables, which store the size of the robot. The stored size is slightly larger than the actual size of the robot. When the LiDAR detects obstacles inside the robot, such as points O2 and O3, it is regarded as detecting the robot itself. These points are automatically ignored. If the detection point is regarded as an obstacle outside the robot, such as points O1 and O4, the point is projected into the map. The flow chart for determining whether a point (taking O1 as an example) is located in a robot in 3D space is shown in Figure 20.

**Figure 21:**
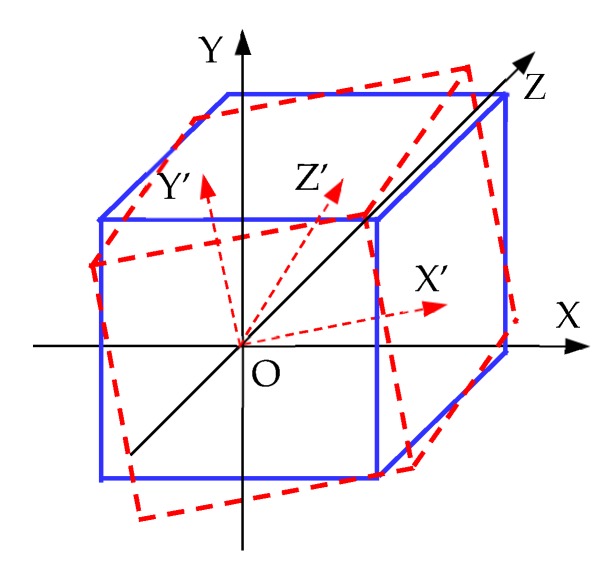
Coordinate correction of the 3D LiDAR.

The results of 3D LiDAR measurements in LiDAR variables are processed. The first step is to determine whether the collision point of the LiDAR is within the range of robotSize1 and robotSize2 variables. If so, this point will be skipped, and the next point will be detected. If not, the point will be corrected, and the coordinate system of the 3D LiDAR itself will be rotated to the same direction as the world coordinate system. As shown in Figure 21, the coordinate system X’Y’Z’ is the self-coordinate system of the 3D LiDAR, and the coordinate system XYZ is in the same direction as the world coordinate system, and the origins of the two coordinate systems are the same. The relative rotation angle of the two coordinate systems is the Euler angle of the 3D LiDAR. The Euler angle used in Unity is in the order ZXY. To transform the world coordinate system into its own coordinate system, X’Y’Z’, it should be rotated *γ* around the Z axis first, then *α* around the X axis, and finally *β* around the Y axis.

After the data members definition is completed, the member functions of the program are defined. A variable for storing the initial position of the robot is defined, and the displacement of the robot from the initial position to the current position, i.e., the 3D vector of the current position of the robot relative to the initial position, is obtained by simulating the output sensor of the inertial navigation sensor on the robot body. Through this 3D vector, the current position of the robot can be obtained, and the localization of the robot can be realized.

The rotation matrix of rotating degree *α* around the X axis is as follows:

$$R_{x}\left(\alpha \right)=\left[\begin{array}{ccc} 1 & 0 & 0\\ 0 & \cos\alpha & -\sin\alpha \\ 0 & \sin\alpha & \cos\alpha \end{array}\right]$$

The rotation matrix of rotating degree *β* around the Y axis is as follows:

$$R_{y}\left(\beta \right)=\left[\begin{array}{ccc} \cos\beta & 0 & \sin\beta \\ 0 & 1 & 0\\ -\sin\beta & 0 & \cos\beta \end{array}\right]$$

The rotation matrix of rotating degree *γ* around the Z axis is as follows:

$$R_{z}\left(\gamma \right)=\left[\begin{array}{ccc} \cos\gamma & -\sin\gamma & 0\\ \sin\gamma & \cos\gamma & 0\\ 0 & 0 & 1 \end{array}\right]$$

The Euler angle of a 3D LiDAR is obtained. It rotates its own coordinate system in the same direction as that of the world coordinate system, contrary to the rotation order mentioned above. It is necessary to first rotate *β* around the Y axis, then *α* around the X axis, and finally *γ* around the Z axis. Thus, the rotation matrix of the Euler angle is as follows:

$$\begin{array}{l} \;\;R_{w}=R_{y}\left(\beta \right)\cdot R_{x}\left(\alpha \right)\cdot R_{z}\left(\gamma \right)\;\\ \;=\left[\begin{array}{ccc} \cos\beta \cos\gamma +\sin\beta \sin\alpha \sin\gamma & -\cos\beta \sin\gamma +\sin\beta \sin\alpha \cos\gamma & \sin\beta \cos\alpha \\ \cos\alpha \sin\gamma & \cos\alpha \cos\gamma & -\sin\alpha \\ -\sin\beta \cos\gamma +\cos\beta \sin\alpha \sin\gamma & \sin\beta \sin\gamma +\cos\beta \sin\alpha \cos\gamma & \cos\beta \cos\alpha \end{array}\right] \end{array}$$

The formula for correcting the detection results of the 3D LiDAR is as follows:

$$V_{w}=R_{w}\cdot V_{s}$$

The result of the modified 3D LiDAR is the three-dimensional vector relative to the center of the 3D LiDAR. The three-dimensional vector of the collision point relative to the center of the robot is obtained by adding the lidarPos variable mentioned above. By adding the results to the three-dimensional coordinates of the robot in the world coordinate system, the three-dimensional coordinates of the collision point in the world coordinate system can be obtained and projected to the map for map reconstruction. Then, the locations of obstacles are judged according to the map information, and the canWalk variable of nodes is updated. Finally, the calculated path is used to control the robot’s movement.

## 4. Simulation Based on Unity3D

### 4.1. Introduction of the Mecanum Wheel Mobile Robot 

#### 4.1.1. Kinematics Model of the Mecanum Wheel Mobile Robot

**Figure 22:**
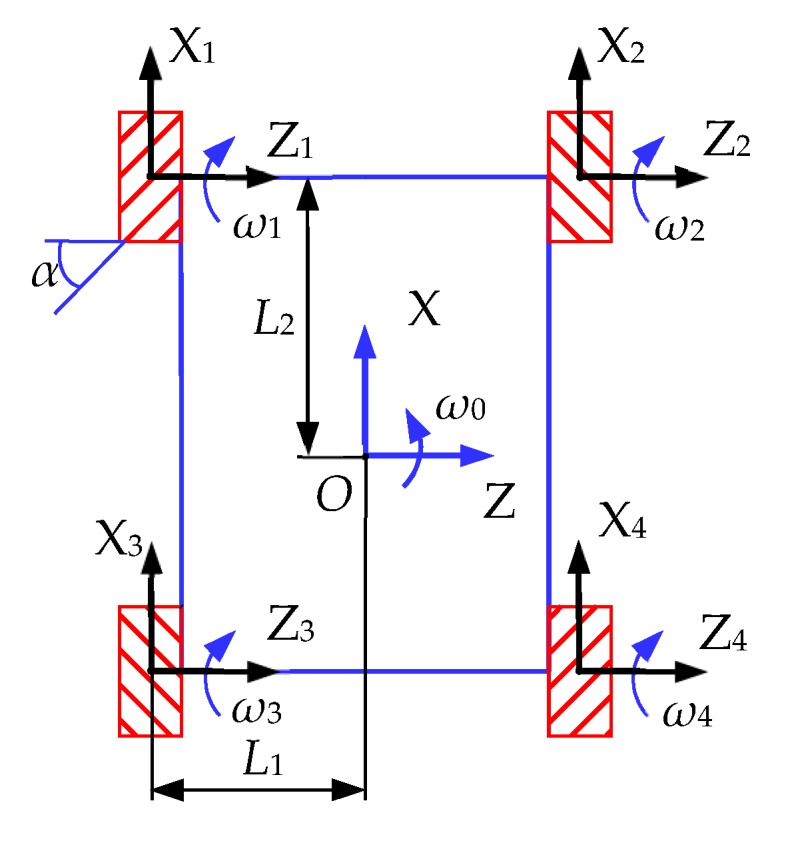
Schematic diagram of the Mecanum wheel robot.

According to the current position of the robot and the position of the nodes on the path, the moving direction of the robot is obtained. According to the kinematic model of the Mecanum wheeled mobile robot, the velocity relationship of the four wheels of the robot is calculated. Finally, according to the speed variable, the velocity of the four wheels is obtained and added to the four wheels of the robot. The arrangement of Mecanum wheels of the Mecanum wheel mobile robot in this paper is shown in Figure 22. The angle *α* between the roller axle and the hub axle is 45 °C

The inverse kinematics equation [58,59] of the system is obtained by kinematics analysis, assuming that there is no slip between the roll and the ground and that the platform moves in the plane:

$$V_{\omega }=J\left(\alpha \right)\cdot V_{O}$$

where, $V_{\omega }={\left[\begin{array}{cccc} \omega_{1} & \omega_{2} & \omega_{3} & \omega_{4} \end{array}\right]}^{T}$ are the speeds of the four wheels. $V_{O}={\left[\begin{array}{ccc} v_{x} & v_{z} & \omega_{O} \end{array}\right]}^{T}$ are the velocities of the robot body in the world coordinate system. J(α) is the Jacobian matrix of the inverse motion equation of the system:

$$J\left(\alpha \right)=\frac{1}{r}\cdot \left[\begin{array}{ccc} 1 & \frac{1}{\tan\alpha } & -\frac{L_{1}\tan\alpha +L_{2}}{\tan\alpha }\\ 1 & -\frac{1}{\tan\alpha } & \frac{L_{1}\tan\alpha +L_{2}}{\tan\alpha }\\ 1 & -\frac{1}{\tan\alpha } & -\frac{L_{1}\tan\alpha +L_{2}}{\tan\alpha }\\ 1 & \frac{1}{\tan\alpha } & \frac{L_{1}\tan\alpha +L_{2}}{\tan\alpha } \end{array}\right]=\frac{1}{r}\cdot \left[\begin{array}{ccc} 1 & 1 & -\left(L_{1}+L_{2}\right)\\ 1 & -1 & L_{1}+L_{2}\\ 1 & -1 & -\left(L_{1}+L_{2}\right)\\ 1 & 1 & L_{1}+L_{2} \end{array}\right]$$

The velocity equations of the four wheels are as follows:

$$\begin{array}{c} \omega_{1}=\frac{1}{r}\left[\begin{array}{c} v_{x} \end{array}+v_{z}-\left(L_{1}+L_{2}\right)\cdot \omega_{0}\right]\\ \omega_{2}=\frac{1}{r}\left[\begin{array}{c} v_{x} \end{array}-v_{z}+\left(L_{1}+L_{2}\right)\cdot \omega_{0}\right]\\ \omega_{3}=\frac{1}{r}\left[\begin{array}{c} v_{x} \end{array}-v_{z}-\left(L_{1}+L_{2}\right)\cdot \omega_{0}\right]\\ \omega_{4}=\frac{1}{r}\left[\begin{array}{c} v_{x} \end{array}+v_{z}+\left(L_{1}+L_{2}\right)\cdot \omega_{0}\right] \end{array}$$

#### 4.1.2. Introduction of the Structure of the Mecanum Wheel Mobile Robot

**Figure 23:**
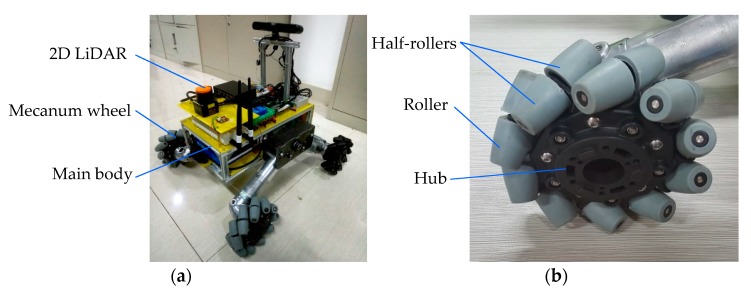
Pictures of the Mecanum wheel mobile robot used in this paper: (**a**) Mecanum wheel mobile robot; (**b**) Mecanum wheel of the robot.

The Mecanum wheel mobile robot used in this paper is shown in Figure 23a. The Mecanum wheel is a kind of intermediate supporting Mecanum wheel, and the roller consists of two symmetrical half-rollers, as shown in Figure 23b. In Figure 22, the distance between the axes of the front and rear wheels is 2*L*_2_ = 400 mm, and the distance between the center lines of the left and right wheels is 2*L*_1_ = 450 mm. The total length and width of the robot are about 550 and 500 mm, respectively.

### 4.2. Establishment of the Virtual Prototype of the Robot

#### 4.2.1. Model Import 

**Figure 24:**
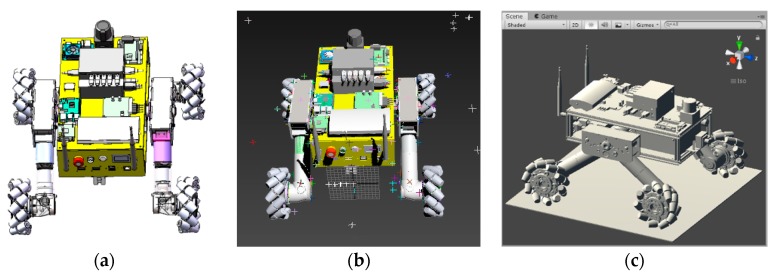
Three-dimensional model of the robot imported from SolidWorks into Unity3D: (**a**) 3D model built in SolidWorks; (**b**) 3D model in 3ds Max; (**c**) 3D model in Unity3D.

The 3D model of the Mecanum wheel mobile robot was built in SolidWorks, and then the file format of the 3D model was transformed into FBX format by Autodesk 3ds Max. Finally, the FBX format model was imported into Unity. The robot models are shown in Figure 24.

#### 4.2.2. Grouping of Model Grids

When the robot model was imported into Unity, the grid of each component was arranged and named in order, which was not conducive to the subsequent addition of constraints and programming, so the imported model needed to be grouped and renamed. In order to facilitate the subsequent addition of constraints and programming, the robot was divided into nine parts, including the main body, four hubs, and small rollers on the four wheels. An empty gameobject named “robot” was created, which was used to store the mobile robot. Nine sub-objects, named body, hub1, hub2, hub3, hub4, wheel1, wheel2, wheel3 and wheel4, were established under the object “robot”. These nine sub-objects corresponded to nine parts of the robot. The corresponding meshes of each part were dragged to the corresponding sub-objects to realize the grouping of the meshes.

#### 4.2.3. Adding the Collider

In the simulation, each roller on the Mecanum wheel was in contact with the collision bodies such as the ground and obstacles. Therefore, it was necessary to use the Mesh Collider component in Unity to add the collision bodies of corresponding shapes to the roller with the Convex selected in the parameter settings.

**Figure 25:**
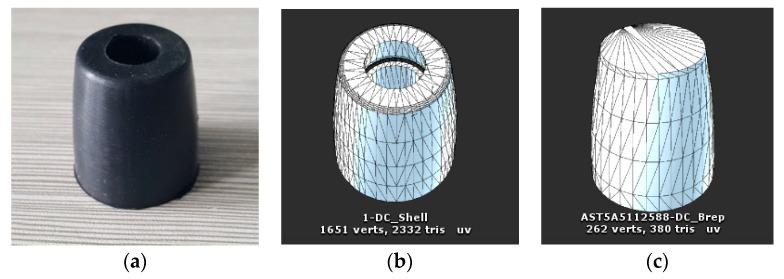
Simplified model of the roller in Unity3D: (**a**) the picture of the roller; (**b**) mesh model of the roller; (**c**) simplified mesh model of the roller.

**Figure 26:**
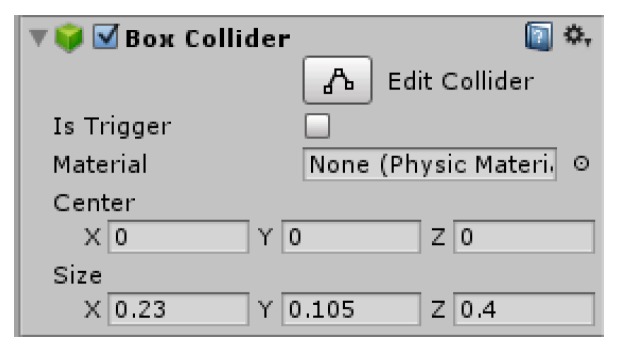
Box Collider component.

The model of small rollers was simplified by removing the hole and corner features in the model, so as to reduce the number of vertices of the model and to avoid errors in establishing the mesh collision body model. Figure 25a is the roller used in the Mecanum wheel. Figure 25b is a mesh model of roller with 1651 vertices, and Figure 25c is a simplified mesh model of small rollers with 262 vertices. The Box Collider in Unity was used to add collision bodies for the rollers and the hub, and the location and size of Box Collider were set. The size was close to the actual size of the robot, but it did not need to be particularly precise, as shown in Figure 26.

#### 4.2.4. Adding Joint Scripts

**Figure 27:**
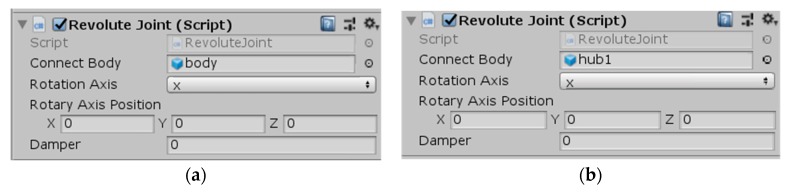
The addition of revolute joint scripts: (**a**) the addition of script to the revolute joint between the body and the hub; (**b**) the addition of script to the revolute joint between the hub and the roller.

In the simulation environment, the robot was only able to move after adding constraints between the various parts of the robot. There were revolute pairs between the hub and the main body and between each roller and the hub of the wheel. The revolute joint script described above needed to be added to these revolute joints. The corresponding parameters in the program settings are shown in Figure 27.

#### 4.2.5. Adding Rigid Body Components

**Figure 28:**
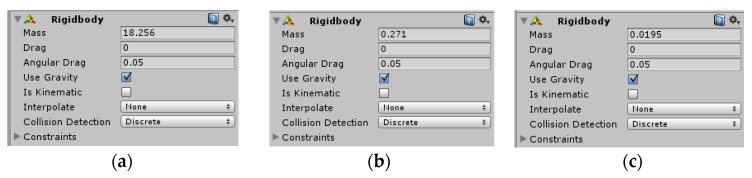
Rigid body components of the roller, hub, and main body: (**a**) rigid body component of the main body; (**b**) rigid body component of the hub; (**c**) rigid body component of the roller.

Rigid body components were added to the main body, hub, and rollers of the object, and the quality parameters of the rigid body were set according to the actual quality of the physical prototype, as shown in Figure 28.

#### 4.2.6. Adding Navigation Algorithm Scripts

The navigation algorithm program was attached to the Unity object, and the variables of the program were connected to the corresponding objects in the Unity environment. In Unity, an empty object named A* was created to add a navigation algorithm program to the object. An empty object named “end” was created in Unity, and the position of the object was the position of the target point of the robot.

**Figure 29:**
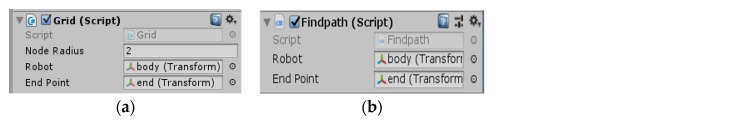

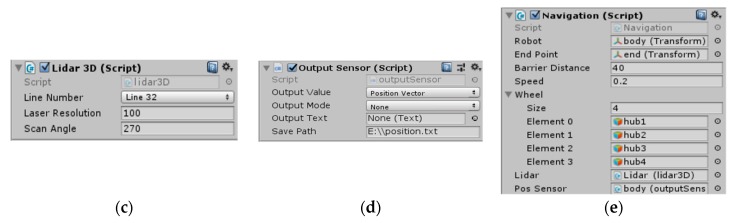
The addition of navigation algorithms: (**a**) the addition of grid script; (**b**) the addition of Findpath script; (**c**) the addition of LiDAR 3D script; (**d**) the addition of output sensor script; (**e**) the addition of navigation script.

Firstly, the “Grid” program was added to the A* object, and the main body of the robot was assigned to the “Robot” parameter. The “end” object was assigned to the End Point parameter, and the value of the Node Radius was temporarily set to 2, as shown in Figure 29a. The smaller the Node Radius value and the smaller the grid, the more accurate the navigation algorithm and the more computational resources it consumes, so the selection needed to be appropriate. Then, the program Findpath was added to the A* object, and the main body of the robot was assigned to the Robot parameter, and the “end” object created earlier was assigned to the End Point parameter, as shown in Figure 29b. Finally, the Navigation program was added to the A* object, the main body of the robot was assigned to the Robot parameter, and the end object was assigned to the End Point parameter. The value of the Barrier Distance was set according to the geometric size of the robot. The “speed” parameter was used to set the moving speed of the robot, where the value of the tentative variable was 0.2. The four hubs of the robot were assigned as “Wheel” parameters.

Next, the LiDAR parameter of the program was assigned. A sub-object named LiDAR under body was created, and its position was adjusted to the position of the robot’s LiDAR. A LiDAR 3D program was added to the LiDAR object and corresponding parameters were set, as shown in Figure 29c. We were able to click the button behind LiDAR parameter in Navigation program and select the LiDAR object to assign parameters. The PosSensor parameter of the program was assigned. The Output Sensor was added to the body object, the Output Value was set as a Position Vector, and the value of the Output Mode was set as none, as shown in Figure 29d. Then, the parameters of the PosSensor of the Navigation program were selected as the body. Finally, the parameters Robot Size 1 and Robot Size 2 were set. Their values represented the size of the robot centered on the LiDAR object, as shown in Figure 29e.

### 4.3. Movement Simulation of the Robot on the Different Ground Types in Unity3D

**Figure 30:**
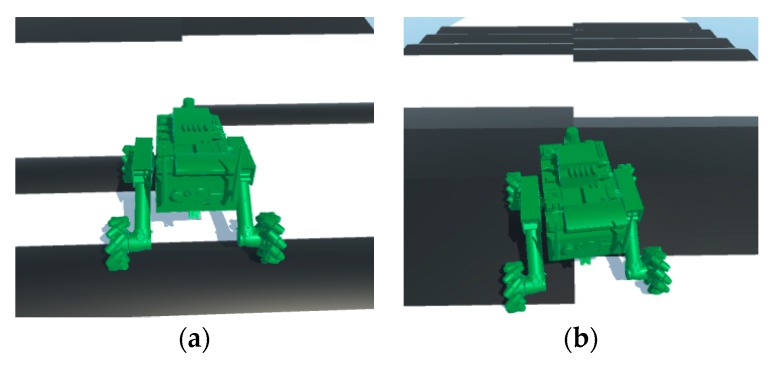

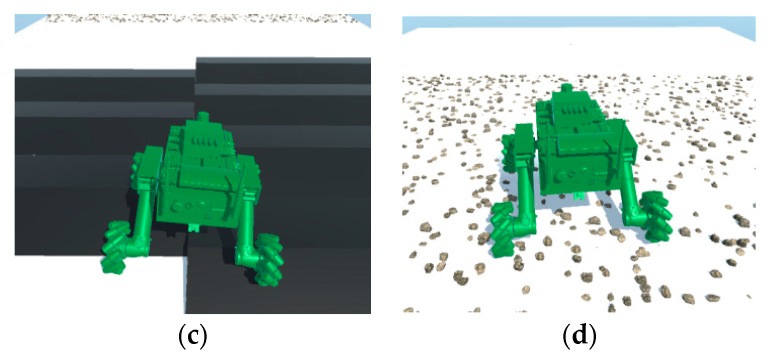
Simulation of the robot passing through the terrain with obstacles presented in Figure 9c: (**a**) passing through round convex obstacles; (**b**) passing through slope steps; (**c**) passing through undulating ground; (**d**) passing through scattered gravel pavement.

**Figure 31:**
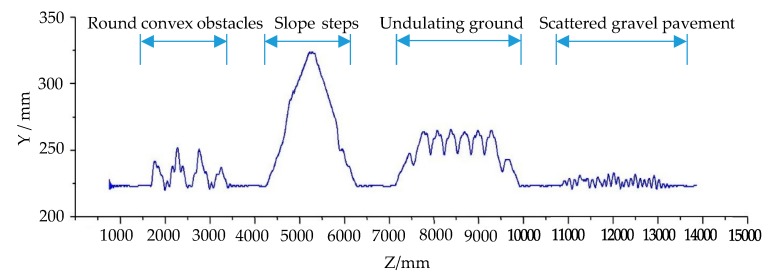
The height trace of the robot while passing through the obstacle terrain simulation.

**Figure 32:**
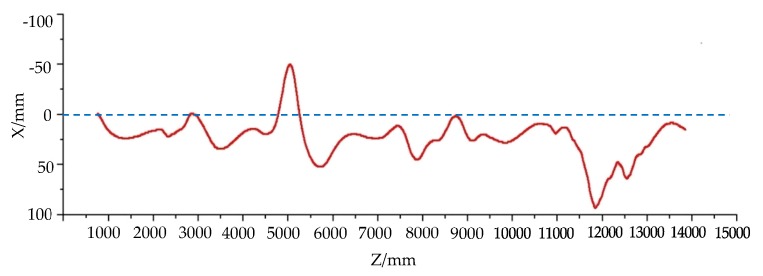
The moving trajectory of the robot on the XOZ horizontal plane in simulation.

When a robot moves on uneven ground, it may slip and the angle of navigation will change, which will affect the movement of the robot and change the route of the robot. Therefore, the movement of the robot should be corrected by turning to adjust the direction of the robot. In this study, the movement of the robot on different ground types was simulated by using the terrain platform of the obstacle simulation, as shown in Figure 9c. The starting point and target point were set on the center line of the simulated terrain. The robot moved along the center line, passing through a cylindrical convex platform, slope step, undulating terrain, and scattered gravel pavement in turn, as shown in Figure 30a–d, respectively. The height trace of the robot when passing through obstacle terrain simulation is shown in Figure 31. This shows the change in the Z coordinate of the robot center during its movement. The curve in Figure 31 reflects the effect of the obstacle on the center height of the robot’s main body. Figure 32 shows the moving trajectory of the robot on the XOZ horizontal plane during the simulation. During the simulation process, the position of the robot was roughly in the vicinity of the centerline of the terrain. However, when the robot passed the obstacle, the moving route of the robot showed different degrees of deviation. The robot corrected itself and returned to the vicinity of the center line. Finally, the robot reached the target point.

In this simulation, the robot did not need to perform obstacle avoidance operations for obstacles on terrain that the robot could overcome. These barrier terrains affected the smoothness of the robot's motion, causing the robot to slip to a certain extent, thus deviating from the planned path, as shown in Figure 32. The simulation results truly reflected the influence of rough and inclined terrain on the motion of the robot in the real scene. In the real process of robot navigation, the influence of terrain cannot be ignored. For example, when the robot moved on the stochastic ground shown in Figure 10a, the uneven terrain will have a certain impact on the robot motion. So, the robot needs perform real-time azimuth adjustment and correct its motion route, which will cause its motion route to have small fluctuations. In this simulation, the influence of uneven terrain on the motion path of the robot was quite obvious, and the robot adjusted its motion in time without affecting the overall trajectory.

### 4.4. Navigation Simulation of the Robot in Unity3D

#### 4.4.1. Navigation Simulation on Stochastic Ground 

In this simulation, the stochastic ground with a pit and a mound shown in Figure 10a was used. There were many small folds on the stochastic ground. Since the Mecanum wheel mobile robot has a poor obstacle-crossing ability when moving laterally, when it moves on uneven stochastic ground, it should avoid lateral movement. So, it was planned that the robot would turn in place at the inflection point of the planned path and continue to move after turning in the direction corresponding to the planned path in the simulation.

**Figure 33:**
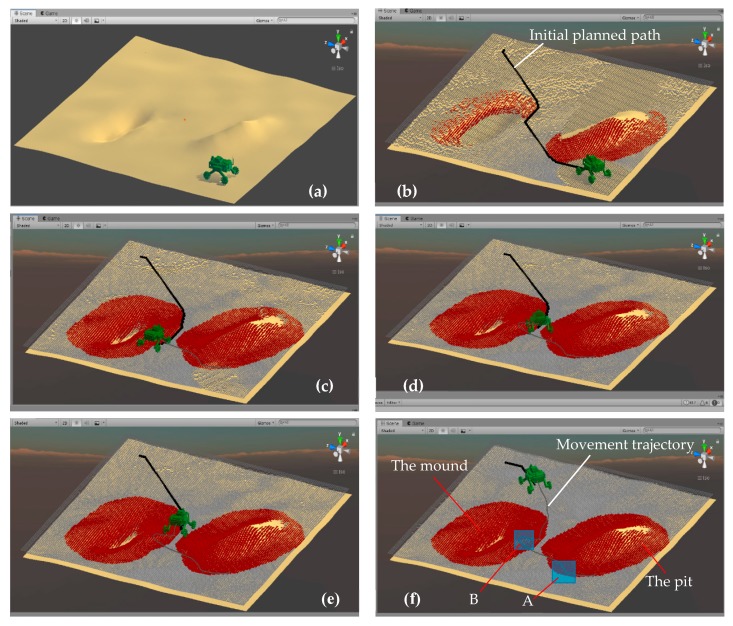
Navigation simulation of the robot on stochastic ground with a pit and a mound: (**a**) the simulated stochastic ground and robot model; (**b–f**) navigation simulation process of the robot.

**Figure 34:**
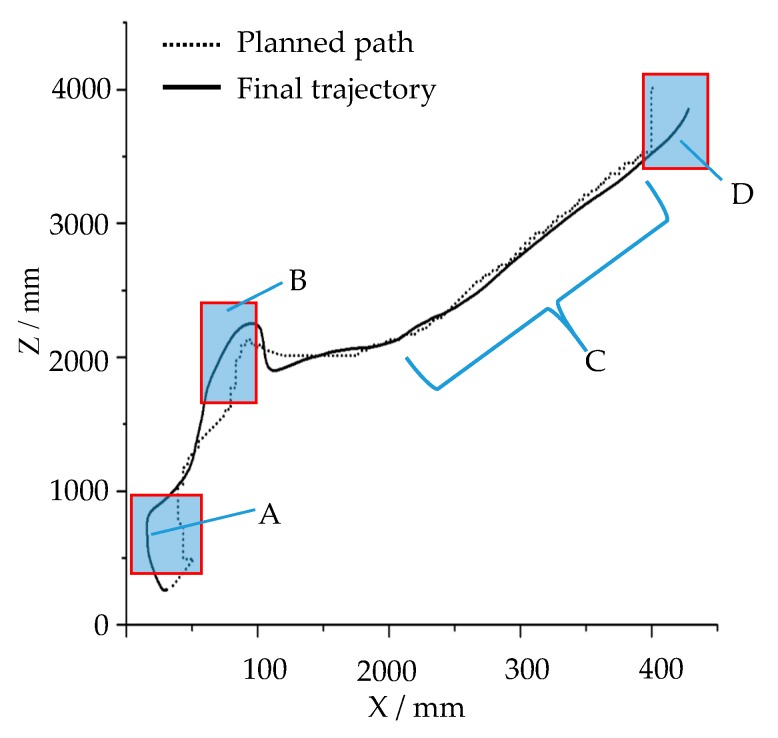
The planned path and final trajectory of the robot in the navigation simulation.

The robot prototype was located at the starting point on the stochastic ground, as shown in Figure 33a. Figure 33b–f shows the navigation simulation process of the robot, where the robot detected the ground surface while constantly moving. The red area was the position of the pit and mound detected by the robot. As the robot moved forward and continued to detect, more and more terrain information was detected by LiDAR, the position of the obstacle was constantly updated, and the red area is expanding. The path calculated and planned by the algorithm is constantly changing. In Figure 33b, the black curve is the initial planned path planned by the algorithm. The robot rounded the mound from one side of the mound and passes through the gap between the mound and the pit and finally reached the target point. The movement trajectory of the robot was recorded and displayed on the screen in the course of motion, as shown in Figure 33f. The changing planned path and the final trajectory of the robot also were recorded in a TXT format file using OutputSensor added to the robot prototype. According to the recorded file, the curve of the planned path and final trajectory of the robot in the horizontal plane were drawn in Figure 34. 

The planned path in the Figure 34 is the path that was constantly revised according to the information of the obstacle terrain as the robot moved and detected. The trajectory of the robot is basically consistent with the planned path curve. In the straight and flat area, the trajectory of the robot coincides with the planned path very well, as shown in section C in Figure 34, but there are still some deviations, such as in segments A, B, and D in Figure 34. 

During the navigation simulation, the robot slides on inclined ground near the pit (A area) and the mound (B area) shown in Figure 33f, so the trajectory of the robot deviates from the planned path. The D part of the trajectory is inconsistent with the planned path, because the inertia causes the robot to move forward at the planned steering position without a timely turn. In general, the path planning of the robot is reasonable, and its trajectory is basically consistent with the planned path, which reflects the credibility of the robot motion simulation. The deviation of the robot's movement trajectory from the planned path reflects that this simulation platform can realistically simulate the influence of the inclined and rough ground on the robot motion. Compared with some simulation platforms, this is one of the advantages of this simulation platform, which can reflect the real interaction between robot and environment. The simulation results can provide predictions for robot motion in real environment and prevent dangerous situations in the process of motion.

#### 4.4.2. Obstacle Avoidance Navigation Simulation of the Robot in a Static Environment

In this navigation algorithm simulation, the plane ground with three obstacle objects shown in Figure 9c, which is a static environment, was used. Since the simulation ground was flat, the robot was able to move along the planned path in two motion modes. The first motion mode was the same as the one that is used in the former navigation simulation. The robot adjusted the direction in situ at the inflection point of the path and then moved forward. In the second motion mode, the robot moved along the planned path while keeping its attitude unchanged.

**Figure 35:**
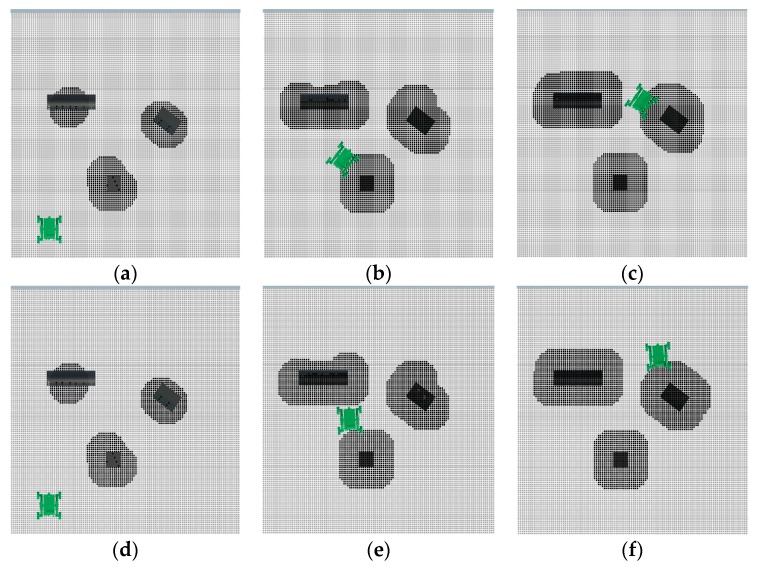
Robot simulation processes on obstacle avoidance ground: (**a–c**) simulation process in the first motion mode; (**d–f**) simulation process in the second motion mode.

**Figure 36:**
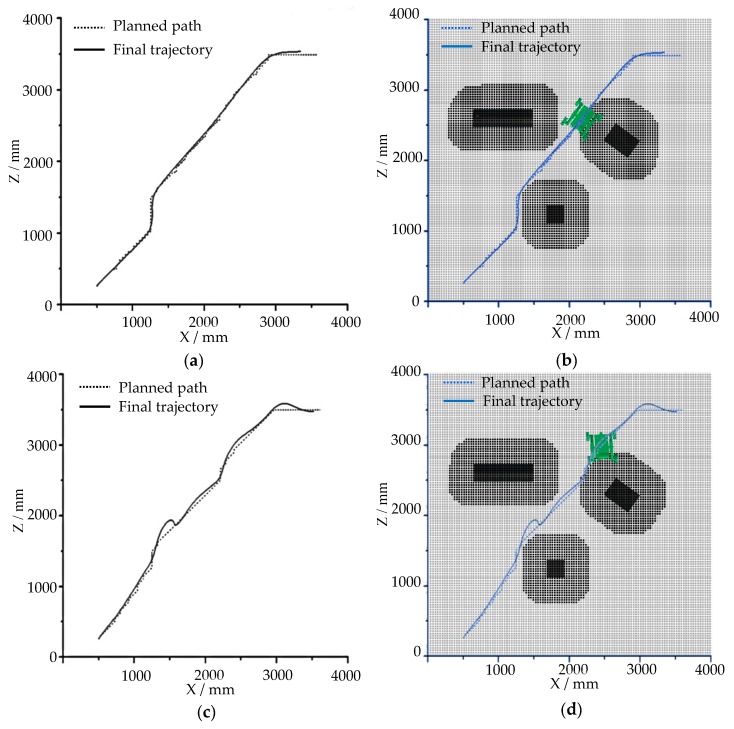
The curves of the planned path and final trajectory of the robot in the horizontal plane in the two motion mode: (**a**) simulation results in the first motion mode; (**b**) combination of the simulation result curves and detected terrain; (**c**) simulation results in the second motion mode; (**d**) combination of the simulation results curves and detected terrain.

The process of robot detection in the first mode is shown in Figure 35a–c, and the process in the second mode is shown in Figure 35d–f. The robot passes through the gap between obstacle 1 and obstacle 2 and the gap between obstacle 2 and obstacle 3 successively and finally reaches the target point. The black area is the position of the obstacle objects detected by the LiDAR on the robot. The curve of the planned path and the final trajectory of the robot in the horizontal plane in the two-motion mode are drawn in Figure 36a,c, respectively. For a clearer understanding of the planned path and the position of the robot trajectory relative to the terrain in the first motion mode, the curve in Figure 36a was combined with Figure 35c, as shown in Figure 36b. The combination picture of the curve in Figure 36c in the second motion mode with Figure 35f is shown in Figure 36d.

Figure 35 shows that the simulation process of the robot matches the obstacle layout in the environment. From Figure 36a,b, it can be seen that the trajectory of the robot was in good agreement with the planned path when the first motion mode was used to simulate the navigation. From Figure 36c,d, it can be seen that when the second motion mode was used to navigate, there was a certain deviation between the robot’s trajectory and the planned path at the turning of the robot, and the overall consistency was good.

#### 4.4.3. Obstacle Avoidance Navigation Simulation of the Robot in a Dynamic Environment

In the practical application of robots, robots usually work in dynamic environments, in which new obstacles may appear, or existing obstacles may change their positions. For example, service robots working in public places may need to adjust their moving routes at any time to avoid collisions with the crowd, and although the environment of factory-inspecting robots is generally static, the entry of personnel or equipment will affect the movement route of the robot. Therefore, the path planning and navigation of robots in dynamic environments is also a focus of current research. A variety of obstacle avoidance algorithms have been simulated, tested, and practiced for different dynamic environments [60,61,62,63]. A common concern in real-time planning is the presence of dead-ends in the state space, i.e., the areas surrounded by obstacles [64]. For example, coal mine rescue robots often encounter dead ends in the search and rescue process. It is important for the navigation algorithm to enable the robot to move out of the dead-ends [65].

**Figure 37:**
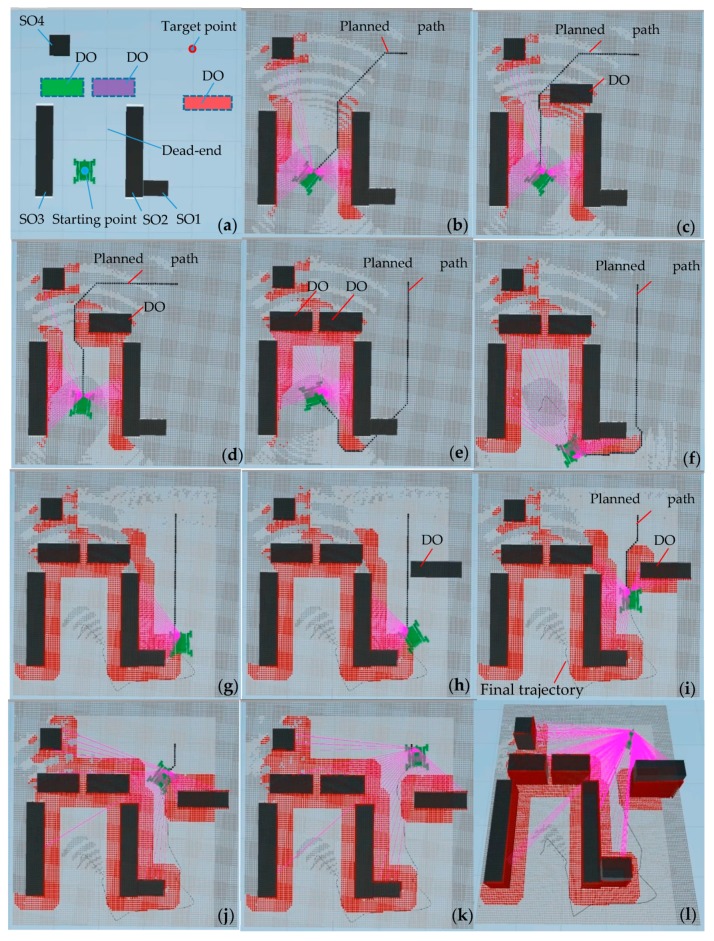
Navigation simulation of the robot in the dynamic environment: (**a**) a dynamic environment consisting of a flat ground, static obstacles (SO), and dynamic obstacles (DO); (**b**) path planning for the environment with static obstacles; (**c**–**d**) path planning after dynamic obstacle DO1 was added; (**e**–**g**) path planning after dynamic obstacles DO1 and DO2 were added; (**h**) the moment when dynamic obstacle DO3 was added; (**i**–**k**) path planning after dynamic obstacle DO3 was added; (**l**) robot arrives at the target point.

In order to simulate a dynamic environment and dead-end form obstacles, this section describes the simulation of an environment consisting of a flat ground, four static obstacles (SO), and three dynamic obstacles (DO), as shown in Figure 37a. In this simulation environment, the four static obstacles SO1, SO2, SO3, and SO4 remain stationary relative to the ground, and the three dynamic obstacles DO1, DO2, and DO3 appear sequentially at intervals. The static obstacles SO2 and SO3 and the dynamic obstacles DO1 and DO2 form a dead-end.

Obstacle avoidance navigation based on improved A* algorithm was simulated in the dynamic environment shown in Figure 37a. In Figure 37b, the robot starts from the starting point, at which time the dynamic obstacles have not been added to the environment, and the first moving path P1 is planned. After the dynamic obstacle DO1 is added, the simulated LiDAR scans to DO1, and the re-planned path P2 is obtained, as shown in Figure 37c. However, the dynamic obstacle DO1 is not completely detected at this time, so there is a superposition of path P2 and obstacle DO1. As the DO1 detection information increases, the path is corrected, and modified path P3 that can bypass DO1 is planned, as shown in Figure 37d. When the dynamic obstacle DO2 is added, the static obstacles SO2 and SO3 form a dead-end with the dynamic obstacles DO1 and DO2, and path P3 is blocked. Then, preliminary path P4, which can guide the robot to move out of the dead end, is planned, as shown in Figure 37e. At this time, path P4 passes through static obstacle SO1 and is modified to path P5 quickly as the robot moves and explores, as shown in Figure 37f. Although DO3 is added but not detected, path P5 has not changed, as shown in Figure 37h. When DO3 is detected, the new path P6 is planned (Figure 37i), and the robot arrives at the target point along path P6, as shown in Figure 37i–l. In order to facilitate the observation, only the laser lines that have detected obstacles were displayed during the simulation.

This simulation shows that the algorithm simulator can implement dynamic planning of the path in dynamic environments and carry out obstacle avoidance navigation simulation. It also verifies that when the robot encounters a dead-end, the robot can re-plan the path that guides it to move out of the dead-end.

**Figure 38:**
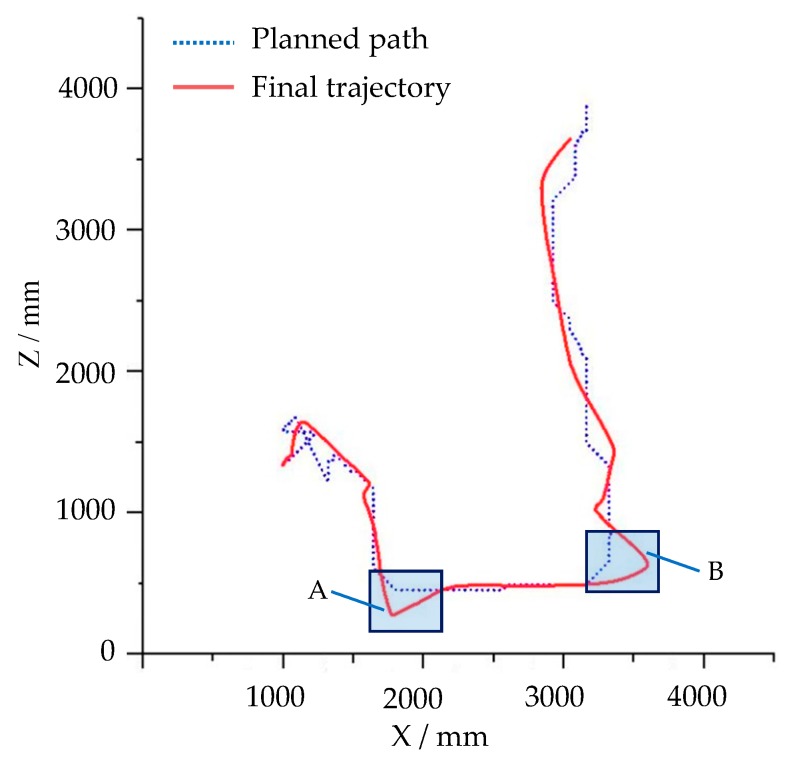
The planned path and final trajectory of the robot in the navigation simulation in the dynamic environment shown in Figure 37.

Figure 38 shows the planned path and final trajectory of the robot in the navigation simulation in the dynamic environment shown in Figure 37. The planned path shown in Figure 38 is the path that is constantly revised as the robot navigates. According to the curves in Figure 38, when the robot makes a large steering angle, the deviation of the robot’s motion trajectory from the planned path is large. There are two large deviation segments of the robot trajectory curve relative to the planned path, segments A and B, as shown in Figure 38. The main reason for this is that there is no real setting of the parameters between the robot’s moving mechanism and the ground. The greater inertia caused the robot to move forward at the planned steering position without a timely turn. The parameters between the robot’s moving mechanism and the ground should be corrected based on the measured data.

## 5. Comparison between the Simulation Result and Test Result in the Physical Environment

In order to evaluate the simulation effect of the simulation platform for robot navigation, judge its feasibility, and evaluate the navigation simulation accuracy, it was necessary to build a navigation accuracy measurement experiment system for the robot in a physical environment. The experimental scheme was as follows [66] : First, using the physical prototype of the robot shown in Figure 23 as the test prototype, the improved A* algorithm was adopted to realize the navigation control of the physical prototype in the created navigation environment. Then, the environment information was tested, and the trajectory of the robot was captured by the Optitrack optical motion capture system of the Natural Company [67]. Finally, the real trajectory of the robot was compared with the trajectory obtained by navigation simulation.

**Figure 39:**
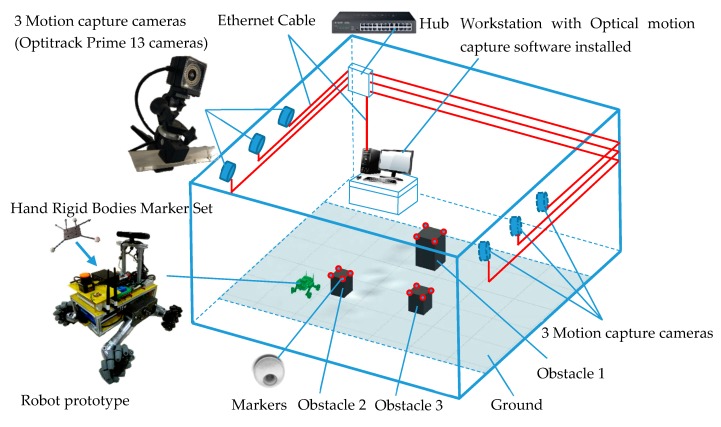
Navigation test system of the robot prototype using the Optitrack optical motion capture system.

**Figure 40:**
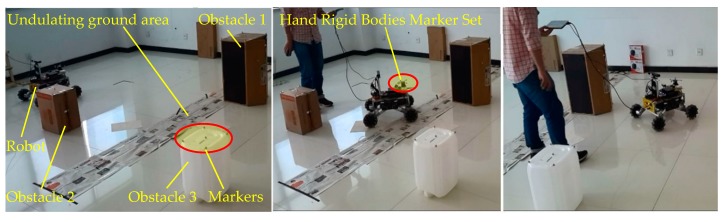
Navigation test of the robot prototype in the physical environment: (**a**) the robot navigates from the starting point; (**b**) the robot moves through the undulating terrain; (**c**) the robot reaches the target point.

The navigation test system of the robot prototype using the Optitrack optical motion capture system is shown in Figure 39. Three Optitrack Prime 13 cameras, high-speed motion capture cameras, were arranged on each side of the test area. The cameras used a Gigabit Ethernet GigE/PoE interface to connect to data and the power supply. All cameras were connected to a Gigabit network Hub with Ethernet cables. An installed workstation with Optical motion capture software named Motive was connected to the hub with a cable. The Motive software was used for the recording, presentation, playback, and remote data services of the position data. The Hand Rigid Bodies Marker Set was fixed on the robot prototype to test the space coordinates of the robot, and the markers were affixed on the obstacles to locate the obstacles in the test environment, as shown in Figure 39 and Figure 40.

**Figure 41:**
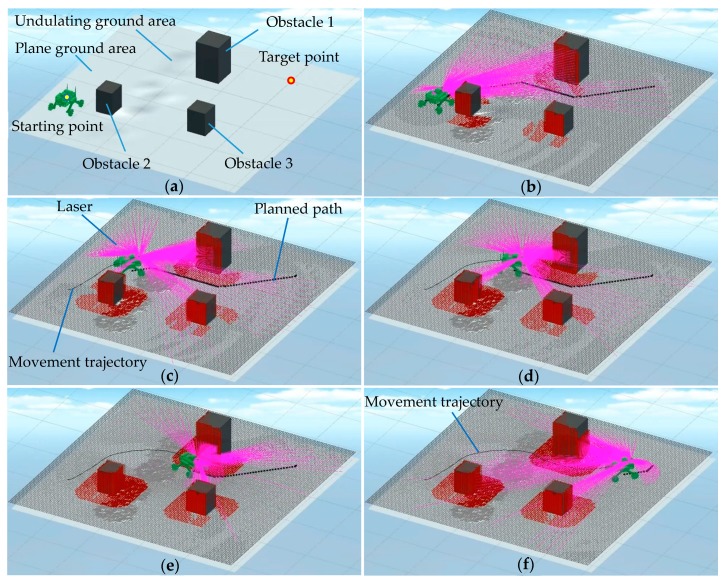
Navigation simulation of the robot in the environment created according to the physical environment in Figure 40: (**a**) the environment created according to the physical environment in Figure 40; (**b**–**f**) navigation simulation process of the robot.

**Figure 42:**
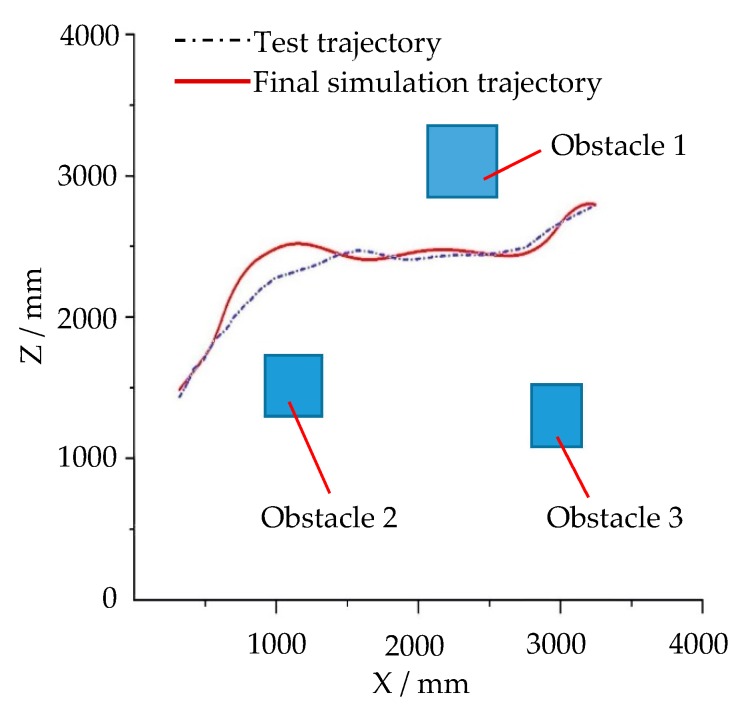
The test trajectory in the physical environment and final simulation trajectory.

In the physical environment shown in Figure 40, a piece of scattered debris was laid on the ground and covered with paper to form an undulating terrain area, and three cubic obstacles were placed. The navigation test process of the robot prototype is shown in Figure 40. The 3D coordinates of the robot were captured and recorded by the Optitrack optical motion capture system, and the 3D test trajectory was obtained. The simulation environment shown in Figure 41a was created according to the physical environment shown in Figure 40. The navigation simulation of the robot in the simulation environment was carried out. The trajectory of the robot virtual prototype in the navigation simulation was recorded in real time in the virtual environment, as shown in Figure 41b–f. The test trajectory and simulation trajectory of the robot are shown in Figure 42. A 2D LiDAR was employed on the physical robot prototype in the physical environment navigation, and a simulated 3D LiDAR was used on the robot virtual prototype in the navigation simulation. Since the ground in the test environment was generally flat and the obstacles were structured cubes, the detection difference between 2D and 3D LiDARs was neglected.

From Figure 42, it can be seen that the test trajectory curve coincides with the simulation trajectory curve substantially. The two curves coincide well in the straight-line section, but the simulation trajectory deviates from the curvature of the measured trajectory as the robot turns. The main reason for the non-coincidence of bending trajectory is the deviation between the physical parameters in the simulation system, such as the friction coefficient and the elastic force, and the actual parameters. In this virtual simulation environment, the inertia of the prototype makes the robot deviate from the planned curve. The physical parameters can be revised according to the actual data of the moving mechanism and ground mechanics. We did not conduct an in-depth study on this. In general the test of robot navigation motion basically verifies the simulation of robot navigation, and the simulation results of the navigation algorithm simulation platform are shown to be credible, and the simulation accuracy is acceptable.

## 6. Conclusions

In this work, after comparing the existing robot simulation platforms or simulators, a simulation platform based on the secondary development of Unity3D was proposed. The virtual prototype of a Mecanum wheel robot and the static and dynamic simulation environments were created, and the A* algorithm was improved for path planning and navigation in unknown 3D environments. A series of navigation simulations of the virtual robot prototype with A* algorithm in different environments were carried out. Using the robot prototype and physical environments, the accuracy of navigation simulation was tested in a navigation measurement system. The simulation results and test results were compared and analyzed. The following conclusions can be drawn from the present study:

(1) Using the simulation platform developed on Unity3D, accurate path planning and navigation simulation in static and dynamic environments can be carried out. In the simulation, the planned paths and motion of the robot were corrected in real time according to the obstacles information detected and the changes of environments.
(2) The improved A* algorithm presented in this paper was validated in three-dimensional unknown environments, which can enable the robot achieve accurate path planning in complex static and dynamic environments, such as the environments with rough terrain, dynamic obstacles and dead-ends.
(3) Taking advantage of the physics engine of Unity3D, the real motion state of the robot and the influence of the ground and terrain on the robot motion can be simulated more realistically in this simulation platform. When the robot passed through rough and inclined terrain, the trajectory of the robot would deviate from the planned path because of the influence of the terrain, which was consistent with the robot motion in physical environments.
(4) Compared with professional simulators such as Gazebo, this method requires scripts to be written for kinematic joints, sensors, the working environments, and so on, which is relatively cumbersome, but once these scripts have been completed, they can be used to simulate different robot prototypes and navigation algorithms. By taking advantage of Unity3D, we can obtain simulation environments which are rich in sensory and physical complexity, and support dynamic multi-agent interactions and good human-computer interaction, which are not well implemented by other current simulators.

This paper presented a method of creating a simulation platform for robot navigation based on Unity3D, and the feasibility and reliability of the platform were demonstrated by simulations and experiments. However, the simulation platform is not perfect. We will further improve the simulation accuracy, human–computer interaction, and the authenticity of the 3D scene. In addition, in order to further verify the simulation platform, we will simulate and test more navigation and obstacle surmounting algorithms and more types of mobile robots, such as tracked and humanoid robots. Based on this study, we are conducting relevant improvements research in a planned manner.

## Figures and Tables

**Table 1:** Comparison of common types of simulation software.

	Unity3D	Gazebo	Webots	V-rep	MRDS	MORSE	Simbad	USARSim	SimSpark
Main Operating System	Windows, Linux, MacOS	Linux	Windows, Linux, MacOS	Windows, MacOS, Linux	Windows	Linux, MacOS	Windows, Linux, MacOS	Windows, Linux, MacOS	Windows, Linux, MacOS
Main Programming Language	C#	C++	C++	C++	C#	Python	Java	C#	C++
Main Physics Engine	PhysX	ODE/Bullet/DART	Fork of ODE	ODE/Bullet	PhysX	Bullet	Built-in	Karma Physics engine	ODE
Java Programming	Yes	No	Yes	Yes	No	Yes	Yes	Yes	No
Can import 3D model	Yes	Yes	Yes	Yes	Yes	Yes	No	Yes	Yes
Physical Fidelity	High	Medium	High	High	High	High	Low	High	Medium
Functional Fidelity	High	Medium	Medium	High	High	High	Low	High	High
Ease of Development	High	Medium	Medium	Medium	Medium	Medium	Low	High	Low

**Table 2:** Definition of variables for 2D laser simulation.

Variables	Type	Function of the Variables
laserResolution		Denotes the resolution of 2D LiDAR, that is, the number of laser lines emitted by the LiDAR in a rotating cycle.
scanAngle	float	Denotes the scanning angle of the LiDAR, that is, the scanning range of the LiDAR.
deltaAngle	float	Represents the angle between two adjacent laser lines, deltaAngle = scanAngle/laserResolution.
result	float	An array that stores the results of radar detection. The size of the array is the value of laserResolution.

**Table 3:** Definition of variables for 3D LiDAR simulation.

Variables	Type	Function of the Variables
lineNumber	int	Represents the line number of the 3D LiDAR, defined as a drop-down selection variable. There is a choice of 4 lines, 8 lines, 16 lines, 32 lines, and 64 lines.
laserResolution		Represents the resolution of 3D LiDAR in the horizontal direction, that is, the number of laser lines emitted by the radar in one revolution.
scanAngle	int	Represents the scanning angle of the LiDAR, that is, the scanning range of the LiDAR.
deltaAngle	float	Represents the angular spacing between two adjacent laser lines in the horizontal direction, deltaAngle = scanAngle/laserResolution
deltaLineAngle	float	Represents the angular spacing between two adjacent laser lines in the vertical direction.
maxLineAngle	float	Represents the angle between the top laser line in the vertical direction and the horizontal plane.
result	float	A two-dimensional array, which stores the results of radar detection.
resultVector	Vector3	A two-dimensional array used to store the 3D vector of the laser collision point relative to the radar.

**Table 4:** 3D LiDAR angles.

Radar Line Number	4 Lines	8 Lines	16 Lines	32 Lines	64 Lines
deltaLineAngle	0.8°	0.8°	2°	1.29°	0.4254°
maxLineAngle	1.2°	2.8°	15°	10°	2°

**Table 5:** The parameters of the test terrain platform.

Obstacle type	Variables	Description of the Obstacles and the Parameters	Values
Scattered gravel pavement	Rang/mm	The edge length of a square scattered gravel pavement	2000
	number	The number of rocks in the square	1000
	Size/mm	The size of the rock in the scattered gravel pavement.	20
	DeltaSize/mm	DeltaSize indicates the fluctuation range of rock sizes in the gravel pavement. By setting DeltaSize, the size and shape of the gravel pavement change, and stochastic generation of the gravel pavement can be realized.	5
Slope step	Glength (*L*_1_/mm)	The slope step is composed of five cubes. The variables representing the shape are shown in Figure 9b. *W* represents the width of the slope step.	500
	Tlength2 (*L*_2_/mm)		500
	Width (*W*/mm)		2000
	Height (*H*/mm)		100
	LeftAngle (α_1_/°)		10/15
	RightAngle (α_2_/°)		10/15
Undulating ground	Number (*N*)	The undulating ground is composed of several triangular prisms, as shown in Figure 9d; *N* represents the number of triangular prisms, and *W* represent the width of the undulating ground.	3/4
	Length (*L*/mm)		300
	Height (*H*/mm)		50
	Width (*W*/mm)		2000
Round convex obstacle	GLength (*L*/mm)	The round convex is a combination of a cylinder and a cube. As shown in Figure 9e, *W* represents the width of the round convex.	600/1200
	Radius (*R*/mm)		300
	Height (*H*/mm)		30
	Width (*W*/mm)		2000/4000

**Table 6:** Definitions of variables of data members from the class Node.

Variables	Type	Function of the Variables
canWalk	bool	Store information on whether the node can pass through a certain area. If the value of the variable is true, the node can pass through; if false, it cannot.
worldPos	Vector3	Stores the 3D coordinates of the node in the world coordinate system.
gridX, gridY	int	Stores the 2D coordinates of the node in the map, that is, the node is located in the gridY row and the gridX column of all the nodes in the map.
maxHeight, minHeight	float	Store the height information of the node and is used to calculate whether the robot can pass through the node.
gCost, hCost and fCost	int	These three variables store the three estimated values of the A* algorithm mentioned above, so as to calculate the target path.

**Table 7:** Variables of data members of the program grid.

Variables	Type	Functions of the Variables
nodeRadius	float	Represents the radius of the node, which determines the spacing of the grid, and the spacing between the two nodes is twice that of nodeRadius.
nodeDiameter	float	Represents the diameter of the node and the value of the variable is twice the nodeRadius.
gridSize	Vector2	Stores the grid area, that is, the map range.
Robot, endPoint	Transform	The two variables store the location of the robot and the location of the target point respectively.
gridCntX, gridCntY	int	The values of gridCntX and gridCntY can be calculated by gridSize and nodeDiameter.
grid	Node	The 2D array, grid [gridCntX, gridCntY], stores the nodes separated by the grid.
path	List<Node>	The path set is used to store the target path calculated in the A* algorithm.

**Table 8:** Definition of data members of the Findpath program.

Variables	Type	Function of the Variables
robot, endPoint	Transform	These two variables are used to store the current location and target location of the robot.
grid	Grid	The “grid” is an object generated by the raster program created above for calling the previous program.
openSet	List<Node>	The openSet corresponds to the OPEN table in the A* algorithm, which is used to store the nodes to be computed.
closeSet	List<Node>	The closeSet corresponds to the CLOSED table in the A* algorithm, which is used to store the computed nodes.

**Table 9:** Definition of data members and related variables in the “Navigation” program.

Variables	Type	Function of the Variables
Robot, endPoint	Transform	Stores the current position and target position of the robot.
barriderDistance	float	Stores the distance of the obstacle expanding outward. The value of the variable is related to the size of the robot.
wheel	Gameobject	The array stores the four wheels of the robot. The program makes the robot move by driving the four wheels to rotate.
speed	float	Represents the moving speed of the robot.
lidarPos	Vector3	Represents the correction of the radar position.
grid	Grid	To call a Grid program.
LiDAR	lidar3D	To call the lidar3D program.
posSensor	outputSensor	To call the outputSensor program.
robotSize1, robotSize2	Vector3	Stores the sizes of the robot.
